# Maximizing Lipidome Coverage of Mouse Liver Following the IV Administration of Gefitinib by Combining Both UHPLC-MS-Based Untargeted and Targeted Lipidomics

**DOI:** 10.1021/acs.jproteome.5c01271

**Published:** 2026-06-13

**Authors:** Robert S. Plumb, Nyasha Munjoma, Lee A. Gethings, Ian D. Wilson

**Affiliations:** † Waters Corporation, 34 Maple St, Milford, Massachusetts 01757, United States; ‡ Waters Corporation, Stamford Ave, Wilmslow SK9 4AX, U.K.; § 170714 Division of Systems Medicine, Department of Metabolism, Digestion and Reproduction, Imperial College,, Burlington Danes Building, Du Cane Road, London W12 0NN, U.K.; ∥ Biochemistry, Cell and Systems Biology, Faculty of Health and Life Sciences, University of Liverpool, Brownlow Hill, Liverpool L69 3GB, U.K.

**Keywords:** liver, untargeted lipidomics, targeted lipidomics, acyl carnitines, free fatty acids

## Abstract

Both untargeted “discovery” and targeted lipid analyses were performed on liver extracts obtained from mice, following the intravenous administration (10 mg/kg) of the tyrosine kinase inhibitor gefitinib, to maximize coverage of the liver lipidome. Untargeted lipid analysis by RP-UHPLC-IM-MS in both +ve and -ve ESI showed time-related changes in lipid profiles, including reductions in the abundances of PCs and PEs and a concomitant rise in the LPCs. Targeted analysis by HILIC-UHPLC-MS using +ve ESI also showed time-related effects on the lipid profiles of gefitinib-dosed mice with PCs, SMs, TGs, and LPCs, particularly for the lipids PC(34:1), PC(32:1), SM(40:1), TG(48:1), and PC(32:0), and effects on a number of acyl carnitines were also noted. In addition, time-related effects were seen using -ve ESI on a range of lipids, including PGs, PCs, LPEs, PEs, and FFAs. The resulting data suggest widespread effects on fatty acid utilization and metabolism may occur in the liver of mice as a result of exposure to gefitinib.

## Graphical abstract

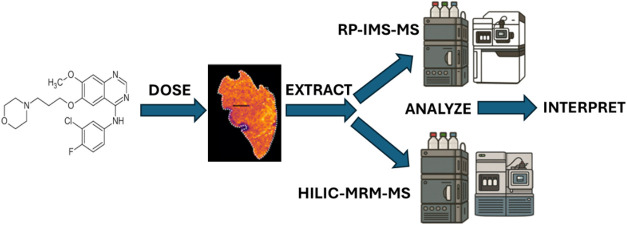

## Introduction

Gefitinib, a tyrosine kinase inhibitor (TKI), initially developed for the treatment of non-small-cell lung cancer,^1^ targets the epidermal growth factor receptor (EGFR). In studies on the pharmacokinetics and metabolic fate of gefitinib in the mouse following both intravenous (IV) and oral (PO) administration^2^ effects on the metabolomes and lipidomes were noted, which appeared to correlate strongly with the plasma concentrations of the drug itself.^3–5^ These effects on metabolite and lipid profiles, implying drug-related effects on both the metabolome and lipidome, may highlight “biomarkers” that can be used to identify time-dependent patient responses (or a lack of them) to drug treatment and monitor efficacy. Such effects may also provide insights into the mode(s) of action(s) (MOA) of the drug itself. They may, in addition, show biochemical responses to drugs that are unrelated to the disease being treated but have effects that might be of value in another therapeutic area, enabling compounds to be repurposed for other conditions.^6^ In addition to repurposing, it might be that by detecting unwanted, “off-target”, interactions with key biochemical pathways, potential toxicological effects could be identified early. An understanding of these pharmacometabodynamic and pharmacolipidodynamic effects may also provide a more complete understanding of a drug’s effects (or lack of them) on the patient and thus aid the implementation of strategies around therapy and personalized medicine. In the plasma-based lipidomic investigation of the effects of gefitinib in male C57Bl/6JRj mice, we observed pharmacolipidodynamic changes in the lysophosphatidylcholines (LPCs), phosphatidylethanolamines (PEs), and phosphocholines (PCs).^4^ In preparation for future studies on gefitinib and related compounds, we were interested in examining the liver lipidome as an organ that represents a major site of lipid biosynthesis. To do this, and to maximize lipidome coverage, we employed both an untargeted “discovery” UHPLC-IM (ion mobility)-MS method^7^ and a quantitative targeted HILIC-MS­(MS) method^8^ on liver extracts from mice obtained at various times following the IV administration of gefitinib at 10 mg/kg.

## Materials and Methods

### Chemicals and Reagents

LC-MS grade water, methanol (MeOH), and dichloromethane (DCM) were purchased from Honeywell (Birkenhead, UK), while LC-MS grade acetonitrile (ACN) and 2-propanol (IPA) were purchased from Biosolve Chimie (Birkenhead, UK). Ammonium formate was purchased from Fisher Scientific (Loughborough, UK). For lipid analysis, the Avanti SPLASH LIPIDOMIX, Odd-Chain LIPIDOMIX, and stable isotope-labeled (SIL) deuterated ceramide LIPIDOMIX Mass Spec Standards were obtained from Avanti (Avanti, Birmingham, AL, USA). Gefitinib was obtained from Sigma-Aldrich (Sigma-Aldrich, Poole, UK). Deuterated gefitinib (d6) and O-desmethyl gefitinib (M523595) were from Cayman (Cayman Chemical, Ann Arbor, Michigan, USA). Leucine enkephalin (LeuEnk) was sourced from Sigma-Aldrich, while sodium formate and the CCS Calibration Mix were from Waters Corp. (Milford, MA, USA). Six CD1 mouse livers were purchased (to provide a blank matrix) from Innovative Research (Peary Court, Novi, MI, USA).

### Mouse Experiments

Details of the animal study design have been described in detail elsewhere.^2^ Briefly, mouse liver samples were obtained during a study on the pharmacokinetics, metabolism, and disposition of gefitinib in male C57Bl/6JRj mice (20–27 g). The study was performed by Evotec SAS (Toulouse, France) after full management review and in strict accordance with both National and EU guidelines and was approved by CEPAL on 08/06/2015 (No. APAFIS#04932.02). The drug was administered by the IV route at 10 mg/kg after formulation in hydroxypropyl-β-cyclodextrin (HPβCD)/acetate buffer pH 4.0 50 mM (10%/90%, w/v). Livers were removed immediately following the sacrifice of two animals at each of 0.5, 1, 3, 8, and 24 h post-dose and frozen immediately (−80 °C) until analysis.

### Internal Standard (IS) Solutions

The lipid IS solution for quantitative targeted lipidomics was prepared by a 250-fold dilution of neat SPLASH LIPIDOMIX. Deuterated ceramide LIPIDOMIX and deuterated gefitinib (d6) (3000 ng/mL) were diluted in IPA:ACN (1:2 (v/v)). The concentrations used for both these and the odd-chain LIPDOMIX standards (dissolved in DCM/MeOH 3:1 (v/v)) are listed in full in the Supporting Information (Table S1A,B).

### Sample Preparation

Lipids were extracted from the mouse liver samples using a modified version of the procedure outlined by Want et al.^9^ Weighed amounts of *ca.* 60 mg (mean 59.1 mg, SD 4.7%, CV 7.96 mg) of liver tissue were placed in 1.5 mL tubes prefilled with silica beads. To this, 1 mL of DCM:MeOH (3:1, v/v) of the SIL IS mixture was added, and the liver was then homogenized using the tissue setting of a Bertin Precellys Evolution (Bertin Instruments, Basingstoke, UK) comprising 3 sessions of 20 s pulses at 6000 rpm with 15 s pauses. After homogenization, 0.5 mL of MeOH: H_2_O (1:1, v/v) was added to obtain phase partition with the tubes centrifuged for 10 min at 2000*g*. The extraction procedure was repeated twice on the remaining pellet to maximize lipid extraction. The 3 extracts were then combined to form the final sample and dried under nitrogen in a fume hood. The dried samples were reconstituted in 1 mL of IPA: ACN (1:2, v/v) with sonication (10 min). Samples were transferred to Eppendorf tubes for a further centrifugation step (5 min, 13,000*g*) to remove particulate matter. The supernatant was then transferred to maximum recovery vials (Waters Corp.) and analyzed by UHPLC-IM-MS analysis for “discovery” untargeted analysis and UHPLC-MS/MS for targeted lipid analysis, respectively.

### Quality Control Samples (QCs)

The untargeted “discovery” lipid analysis was monitored using pooled quality control samples prepared by mixing 5 μL aliquots of all the reconstituted extracts from the liver sample preparations described above to create a pooled QC (study reference). In addition, QCs were prepared by mixing 5 μL aliquots of blank matrix CD1 mouse liver extracts (blank matrix QC) and liver extracts from gefitinib-dosed mice (study QC) (for references to the preparation and use of QCs, see refs 10–12 ). All 3 QC sample types were acquired at regular intervals (at the beginning of the run and every 8 sample injections thereafter) to monitor the performance of the analytical system and check for analytical drift during data acquisition.

### Calibration Curve Preparation for Targeted Quantitative Analysis

Calibration curves were prepared using six CD1 mouse liver extracts (prepared as described above), reconstituted in IPA/ACN containing the Odd-Chain LIPIDOMIX at six concentrations, ranging from 5 ng/mL (LPE) to 84,750 ng/mL (cholesterol ester). Gefitinib and the O-desmethyl metabolite were also included in the calibrant solutions at concentrations ranging from 12 ng/mL to 1333 ng/mL (with quantification performed as part of the targeted lipidomics profiling). Samples were transferred to Eppendorf tubes for a further 5 min centrifugation step to remove debris. The supernatant was transferred to total recovery vials (Waters Corp.) for LC/MS analysis. As described above, the concentrations (ng/mL and μg/mL) of the calibrants are provided in Table S1A,B.

### Sample Analysis

The gefitinib liver extracts were randomized for analysis and were interspersed with blank matrix samples throughout the assay. Each of the gefitinib mouse liver extracts was analyzed in triplicate in both untargeted and targeted assays, including blank matrix, QC, and calibration samples, a total of 152 samples in each analysis (148 excluding blanks). The samples were then randomized, and the same run order was used for both positive (+ve) and negative (-ve) electrospray ionization (ESI) modes of analysis. The sample run lists for the untargeted and targeted analyses are provided in Table S2.

### Untargeted Discovery Lipidomic Profiling by (RP) UHPLC-IM-MS

Discovery lipidomic analysis was performed using reversed-phase (RP) UHPLC-IM-MS in both +ve and -ve ESI.^7^ A Waters ACQUITY Premier UPLC chromatography system, equipped with a binary solvent manager and flow-through needle sample manager (Waters Corp.), was used for the chromatographic separations. The UPLC system used a 2.1 mm × 100 mm ACQUITY Premier CSH C18 1.8 μm chromatography column (Waters Corp., Milford, USA), maintained at 55 °C. Following sample introduction (0.5 μL +ve ESI and 2 μL -ve ESI), the column was eluted using a multilinear solvent gradient (Table 1) formed from 10 mM ammonium formate in 60:40 v/v ACN:H_2_O with 0.1 formic acid (solvent A) and 90:10 v/v IPA/ACN 10 mM ammonium acetate with 0.1% formic­(solvent B) at a flow rate of 0.4 mL/min. The column effluent was monitored using an IM-MS workflow operated on a Synapt XS ion mobility-enabled mass spectrometer (Waters Corporation, Wilmslow, UK) over the mass range 50–1200 *m*/*z* with the low-energy (MS1) collision energy voltage set to 4 eV. The elevated collision energy (MS2) data (used to provide fragment ions) were obtained via a linear ramp from 17 to 40 eV. LeuEnk (200 pg/μL), infused at 20 μL/min with an acquisition interval of 30 s, provided a lockspray signal *m*/*z* = 556.2771 (+ve):554.2615 (-ve). The capillary voltages were 2.5 kV for +ve ESI and 1.7 kV for -ve ESI. A cone voltage of 20 V and a source temperature of 150 °C were used. Nitrogen gas flow rates of 20 L/h for the cone gas and 1000 L/h for the desolvation gas were employed with the desolvation temperature set to 600 °C. The ion mobility settings consisted of a T-wave velocity of 650 m/s with a pulse height of 40 V. The drift gas for IM was nitrogen (180 mL/min), and calibration over the collision cross section (CCS) range of 130–306 Å^2^ was achieved using the Major Mix IMS calibration kit. The calibration of the TOF over the acquisition mass range was performed with 0.5 mM sodium formate with MS data collected using MassLynx *vs* 4.2 software (Waters Corporation, Wilmslow, UK).

**Table 1:** RP-UHPLC Conditions

time (mins)	flow (mL/min)	% *A*	% *B*
initial	0.4	99	1
2	0.4	80	20
5	0.4	50	50
5.1	0.4	99	1
8	0.4	99	1

### Targeted Lipidomic Analysis by HILIC-MS

Targeted lipidomic and gefitinib analyses were performed using HILIC coupled to a tandem quadrupole mass spectrometer operated in both +ve and -ve ESI^8^ for lipids and +ve ESI for gefitinib and its metabolites M523595 (O-desmethyl gefitinib (*m*/*z* = 433.1 → 128.1)) M3 (cleavage of morpholine group followed by carboxylic acid formation (*m*/*z* = 392.1 → 318.0)) and M6 (M537194) (*m*/*z* = 421.1 → 197.7). The separations were performed using a Waters ACQUITY Premier UPLC system as described above (Waters Corp.), configured with a 2.1 mm × 100 mm 130 Å, 1.7 μm ACQUITY Premier BEH Amide chromatography column maintained at 45 °C. Separations were performed using a multilinear gradient formed from 95:5 (v/v) ACN:10 mM ammonium acetate (solvent A) and 50:50 (v/v) ACN:10 mM ammonium acetate (solvent B) (see Table 2) delivered at 0.6 mL/min. Sample extracts (1 μL) were analyzed using HILIC-MS/MS with data collected from 0 to 5 min. Analytes were detected using multiple reaction monitoring (MRM) with a Xevo TQ-XS tandem quadrupole mass spectrometer (Waters Corp., Wilmslow, UK). Source and desolvation temperatures were set at 500 and 120 °C, respectively, with capillary voltages of 2.8 kV in +ve and 1.9 kV in -ve ESI, respectively. Desolvation and cone gases (nitrogen) were set to 1000 and 150 L/h, respectively, while the collision gas flow was 0.13 mL/min. The MRM transitions employed were those described in ref 8. The instrument was operated by, and data were collected, using MassLynx version 4.2 software (Waters Corp. Wilmslow, UK).

**Table 2:** HILIC-UHPLC Conditions

time (mins)	flow (mL/min)	% *A*	% *B*
initial	0.6	99	1
2	0.6	80	20
5	0.6	50	50
5.1	0.6	99	1
8	0.6	99	1

### Data Analysis

The untargeted LC-MS data were aligned, normalized to “sum of areas” (also known as “sample sum” or total area normalization), and peak-picked using Lipostar (Molecular Discovery, Barcelona, Spain). The processed data were further interrogated using a variety of statistical analysis tools embedded in Lipostar^13^ (and MetaboAnalyst^14^). An acceptance criterion was applied to the data such that only features (*m/z-*t_R_ pairs), in either +ve and -ve ion modes, that had a CV <30% were accepted for statistical analysis. Analysis of the targeted lipidomic data was performed using both TargetLynx XS ver. 4.2, (Waters Corporation, Wilmslow, UK) and Skyline (MacCoss Lab, WA, USA) as described in Want et al.^9^ The SIL standards of deuterated ceramide LIPIDOMIX and SPLASH LIPIDOMIX were used as internal standards based on the corresponding natural abundance class of the lipid. MetaboAnalyst software (https://wwwmetaboanalyst.ca/, September 2025) was used to perform multivariate statistical analysis (MVA) on both untargeted and targeted data (*e.g*., see Figures 1, 3, 4, and Supporting Figures S2,4–6,12,13–15). MetaboAnalyst uses visual diagnostics from preprocessing, which are widely used in metabolomics to assess and correct normality or homoscedasticity of data. The MetaboAnalyst workflow includes Data Processing → Normalization/Transformation → Data Quality Check. The MVA tools employed in this work included unsupervised principal component analysis (PCA) and supervised partial least-squares discriminant analysis (PLS-DA). Supervised models were subject to validation testing via permutation tests (1000), where evidence of a real effect was indicated by *p* < 0.001, and cross-validation methods (5-fold CV).

**Figure 1:**
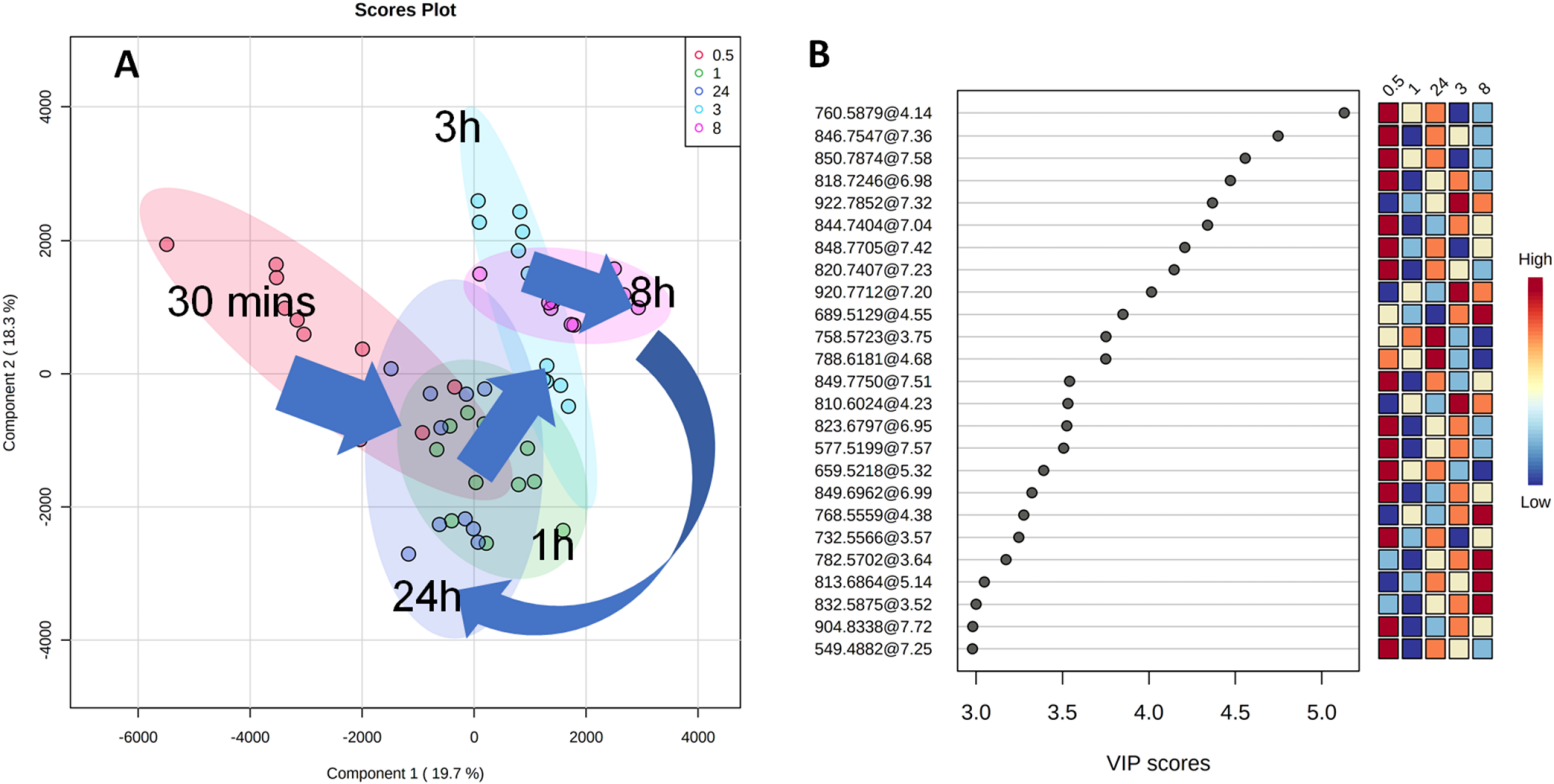
PLS-DA of RP-UHPLC-IM-MS qualitative “discovery” gefitinib +ve ESI data (0.5–24 h post dose) excluding drug-related features and variables in less than 50% of the sample groups, interquartile range filtering (IQR), and Pareto scaling. (A) Scores plot and (B) the top 25 features by variable importance in projection (VIP) (**PCA score plot** provided in Figure S4, see also Table S3 for putative identities).

### Lipid Identification

For the untargeted analysis, identification was performed using the Lipostar identification workflow as discussed in detail by Goracci et al.^13^ For this study, an in-house Lipidomic Profiling CCS library, containing predicted CCS ^TW^CCSN_2_ values for over 3200 lipids,^16^ and LIPIDMAPS database (containing data for over 40000 lipids, https://www.lipidmaps.org/ were accessed on a number of occasions for this work, most recently July 2025) were used for lipid identification.^15^ The mass error tolerances used were 5 ppm for the precursor and 10 ppm for the fragments, with the CCS values within 5% of the predicted values. A final overall score of at least 60, resulting from a combination of the mass score, isotopic pattern score, fragment score, and CCS score, was used to provide high-confidence identifications. The scoring system is discussed in the Supporting Information provided by Goracci et al.^13^

## Results and Discussion

In this study, a two-step approach was employed for the analysis of the mouse liver lipid extracts in order to maximize data recovery from the samples. The initial analysis of the liver extracts was conducted using an untargeted “discovery” method, based on UHPLC-IM-MS, to profile lipids. By their very nature, such discovery methods detect significantly more features than targeted methods, but are at best semiquantitative (providing fold changes, etc., based on relative peak intensities). This untargeted method was aimed at providing a wide coverage of lipids of all classes and was, as far as possible, unbiased in what was detected. Following untargeted lipidomics, a quantitative HILIC-MS/MS class-based targeted method for lipid analysis was employed. In addition, the targeted method was used to determine the amounts of gefitinib and also the relative amounts of any gefitinib metabolites detected in the liver extracts (see below).

### Discovery Untargeted RP-UHPLC-IM-MS-Based Lipidomics of Liver Extracts

Typical base peak chromatograms (BPI) for both +ve and -ve ESI MS mouse liver sample extracts obtained by untargeted RPLC-IM-MS (HDMS^e^) are provided in Figure S1A,B, respectively. Visual inspection of these data showed that the lipids were well resolved with the polar lysophospholipids (LPC), lysophosphatidylserines (LPS), and free fatty acids (FFA) eluting at the beginning of the gradient (0.6–1.4 min). Other polar lipids, such as the phosphocholines (PC), phosphatidylethanolamines (PE), sphingomyelins (SM), and ceramides (Cer), eluted between 2.8 and 5.4 min, and the mono-, di-, and triglycerides (TG) eluted with the cholesterol esters between 5.7 and 8.0 min. Analysis of the study reference QC sample data showed that there was no observable analytical drift in the data set, while technical replicates showed good reproducibility of peak response for both +ve and -ve ESI MS. The +ve and -ve LC-IM-MS data were then filtered to remove isotopic interferences, and only features meeting the required acceptance criteria of CV < 30% and detected in more than 50% of the study reference QC samples were accepted for further statistical analysis. Based on this curation of CV and replication, a total of 1544 and 1562 lipid features were detected in the study reference QCs for +ve ESI and -ve ESI, respectively. Using additional thresholding, which included mass tolerances (<5 ppm (precursor) and <10 ppm (fragments)), the presence of fragment ions (>2 fragment ions) and isotope similarity, resulted in *m*/*z*-t_R_ features being assigned with high confidence reduced to 215 +ve ESI+ and 184 -ve ESI- lipids. These identifications were merged in Lipostar to report a single lipid species based on adducts from each polarity and retention time alignment to leave only 303 total identifications, meaning 96 lipids were common to both ESI modes. Initially, these data were analyzed using PCA, as were all subsequent data sets generated in this investigation. If group separation was observed using PCA, as seen for, *e.g*., the untargeted +ve ESI data (Figure S2), PLS-DA was used for further MVA as it provided a reduction in the data dimensionality and therefore superior feature detection. As Figure S2 shows, both unsupervised and supervised MVA of the +ve ESI data showed similar separations for the matrix control and IV gefitinib samples, with the study reference QCs again clustering between the two groups. Some of the features detected by MVA, eluting between 0 and 0.7 min, corresponded to gefitinib and metabolites M3 (cleavage of the morpholine group followed by carboxylic acid formation) and M6 (M537194 ring-opening and partial degradation of the morpholine ring).^2^ The maximum observed amounts of gefitinib and metabolite M3 were seen in the 0.5 h post-dose liver extracts (and M3 was still detectable in the 8 h post-dose samples) (Figure S3). In contrast, M6 increased in amount from the 0.5 h time point, reaching its highest observed concentration at 3 h post dose, and was still present in small amounts in the final 24 h liver extract (Figure S3). However, given that the extraction procedure was not optimized for drug metabolite extraction, but for lipids, the metabolite coverage provided by this method is almost certainly incomplete. While they were not statistically significant contributors to the separation, these drug-related features were nevertheless eliminated from further analysis of these data in order to concentrate solely on changes in the lipid profile of the liver.

Following the elimination of the drug-related signals, the PLS-DA of the normalized +ve ESI MS data from the gefitinib-dosed group showed the separation of the individual sampling occasions, with PC1 and PC2 accounting for *ca.* 40% of the observed variation (Figure 1). As can be seen from this figure, there was an apparent, time-related, trajectory from the 0.5 sample through the 1, 3, 8, and 24 h samples. In the case of the +ve ESI data shown in Figure 1, a PLS-DA model with 5 components achieved high cross-validated performance (*R* ^2^ *Y* ≈ 0.95–1.00, *Q* ^2^ ≈ 0.90, CV accuracy ≈ 0.90). The *R* ^2^–*Q* ^2^ gap was small (<0.1), indicating limited overfitting. A 1000-iteration permutation test yielded *p* < 0.001 (0/1000), confirming that model performance substantially exceeded that expected under random class assignment (see Figure S4).

The top 25 lipid signals (after filtering to remove adducts (NH_4_^+^, Na^+^, K^+^) and isotopes, etc.), with the highest variable importance in projection (VIP) scores, were annotated and identified, wherever possible, using a variety of approaches as described in the Materials and Methods Section. The 25 lipids having the highest VIP scores in the +ve ESI data included 6 PCs, 13 TGs, 1 SM, 1 PE, 3 DGs, and 1 PA (see Table S3 for lipid identities). The lipid signals contributing most significantly to the observed variation in these untargeted +ve ESI data were *m*/*z* 760.5879, t_R_ 4.14 min (PC 34:1); *m*/*z* 846.7547, t_R_ 7.36 min (TG 50:3); *m*/*z* 850.7874, t_R_ 7.58 min (TG 50:1); *m*/*z* 818.7246, t_R_ 6.98 min (TG48:3); and *m*/*z* 922.7852, t_R_ 7.32 min (TG 56:7).

Following initial MVA of the untargeted -ve ESI data using PCA, further statistical analysis by PLS-DA showed that gefitinib was a significant contributor to the observed variation in these data, and, as with the +ve ESI data, spectral features for both gefitinib and its metabolites were excluded from the data modeling. Scores plots for both the PCA and PLS-DA are provided in Figure S5 together with the *R* ^2^ and *Q* ^2^ and permutation test results for PLS-DA. Having removed drug-related signals, the PLS-DA analysis of the untargeted -ve ESI data (Figure S6) showed that PCs 1 and 2 accounted for *ca.* 30% of the variance in these data, respectively, and also provided a clear time-related trajectory moving from 0.5 h through the 1, 3, 8, and 24 h liver samples. As with the +ve ESI MS analysis, the top 25 lipid signals contributing significantly to the -ve ESI PLS-DA data (Table S3) were investigated using the same databases and approach to assign lipid identities. These lipids were made up of 10 Cer’s, 5 PEs, 4 PCs, 1 PG, 1 SM, 1 FFA, Glycinprenol-9, and 2 unidentified lipids (Table S3). The lipid signals which contributed most significantly to the observed variation in the -ve ESI data were related to the features labeled *m/*z 680.62, tR 6.23 min (Cer­(d40:1)); *m/*z 694.63, tR 6.44 min (Cer­(d42:1)); *m/*z 766.54, tR 4.48 min (PE(38:4)); and *m/*z 610.54, tR 4.94 (Cer­(d36:1)).

When the top 25 VIP scores from the +ve and -ve ESI MS data were combined (see Table S3), it showed that the lipids contributing most to the observed variance in the analysis were composed of 10 PCs, 13 TGs, 10 Cer’s, 6 PEs, 1 PG, 1 PA, 1 SM, 3 DGs, 1 FFA, glycinprenol-9, and 2 unidentified lipids. The t_R_, CCS, and *m*/*z* data for the top 25 lipids detected in both ionization modes are provided in Table S3.

While recognizing that the discovery profiling method was not quantitative, it nevertheless provided useful information on changes in the relative abundance of these lipids. It is also interesting to note that the PCs (phosphatidylcholines) appeared to decrease in relative abundances after drug administration (*e.g*., PC(34:1) and PC(36:4)), reaching a minimum in the 3 h post dose liver sample extracts, before increasing again by 24 h post dose (Figure S7). PCs can be converted to *e.g*., LPCs via the action of phospholipase A2 (PLA2) and lecithin acyltransferase (LCAT) via the Lands cycle.^17^ Examination of data for the LPC (which do not feature in the top 50 lipids listed in Table S3) showed that, as might be expected, in contrast to the PCs, the LPCs appeared to increase in abundance initially following drug administration, peaking between 3 and 8 h post dose (*e.g*., LPC(20:4) and LPC(18:2)). The relative amounts of these LPCs then declined again to a minimum at 24 h post dose (Figure S7). This inverse relationship between PC and LPC abundances following gefitinib administration suggests that the drug and/or its metabolites may indeed have had an effect on the balance between PC and LPC.

### Gefitinib and Related Metabolites

Liver drug concentrations of gefitinib were estimated with a “fit for purpose” HILIC-MS/MS assay performed as part of the targeted lipidomic analysis described below. Although the assay was not fully validated for gefitinib, the stable response of its d6-IS throughout the analytical batch, as shown in Figure S8A, provides some reassurance in the validity of the results. This figure also shows the variation in gefitinib responses in the randomized liver extracts, and a mass chromatogram for an extracted liver study QC sample is provided in Figure S8B for gefitinib and gefitinib d6 (a standard curve is provided for gefitinib as part of Figure S9). Peak observed drug concentrations in these extracts were observed, as would be expected for an IV dose, in the 0.5 h liver samples, with a *C*_max_ of *ca.* 15,000 ng/mL (corresponding to *ca.* 300 ng/mg of tissue). Thereafter, concentrations in the liver extracts declined, reaching *ca.* 3500 and 90 ng/mL at 8 and 24 h post dose, respectively (corresponding to 70 and 2 ng/mg of liver tissue), as detailed in Table S4 and illustrated in Figure 2A,B, respectively. The relationship between the plasma^2^ and liver extract concentrations for gefitinib is illustrated in Figure 2B and indicates that, as would be expected, liver concentrations of gefitinib and those of plasma generally appeared to be correlated and declined at similar rates.

**Figure 2:**
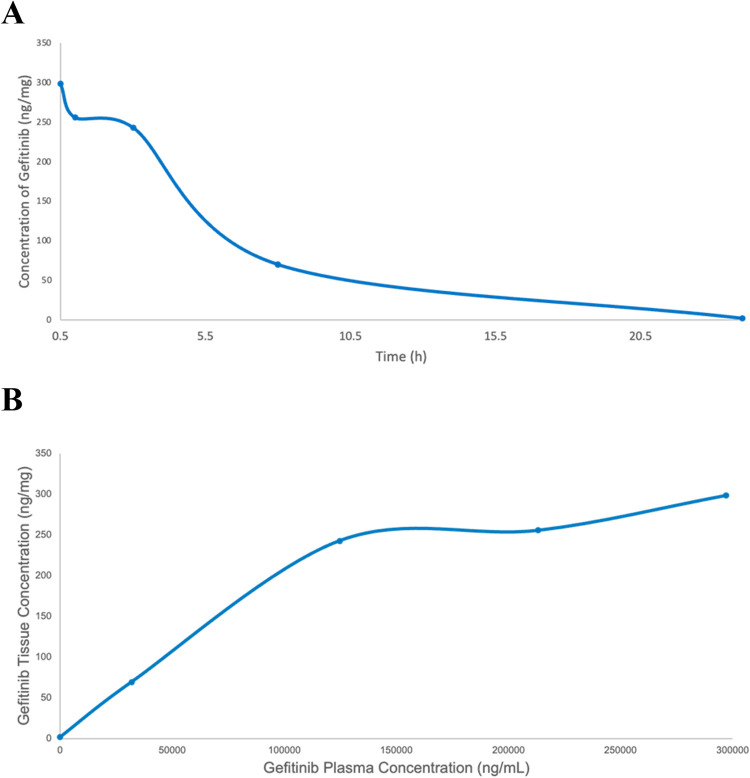
(A) Concentrations of gefitinib in liver extracts following IV administration at 10 mg/kg. (B) Liver concentrations of gefitinib in liver extracts *vs* plasma following IV administration at 10 mg/kg.

The O-desmethyl metabolite of gefitinib (M523595) was only detectable in liver extracts at 0.5 and 1 h post dose, and while present, it was below the limit of quantification. Two other metabolites, M3 and M6 (see Molloy et al.^2^), were also detected in these liver extracts, with the former showing a similar profile to gefitinib while M6 peaked in the 3 h samples (Figure S3). However, in the absence of standards, no attempt was made at the quantification of either M3 or M6.

### Quantitative Targeted Lipid Analysis

While typically providing reduced coverage compared to untargeted discovery methods in terms of the number of “features” detected, if used with appropriate calibrants, targeted lipidomic methodologies benefit from producing identities and quantitative data for the preselected analytes. To investigate the changes in lipid concentrations following gefitinib dosing selected lipids were measured in the liver extracts using the HILIC-MS/MS-based “LipidQuan” method.^8^ HILIC, unlike the RPLC discovery separation used for the untargeted analysis above, separated lipids on the basis of their polar head groups. This class-based separation (see Figure S9 and B), when combined with SIL-labeled standards and an odd-chain lipid mixture, can be used to provide class-based quantitative data.^18^ This approach, originally developed and validated for plasma/serum, was combined with an established liver extraction procedure^9^ and used as a “fit for purpose” method for these mouse liver samples. The lipids subject to analysis included not only some of those highlighted in the VIP lists from the discovery analysis described above, but many additional examples, thereby increasing their coverage from the same classes. Calibration curves for these lipid classes in mouse liver extracts were linear over the range 5 ng/mL (LPEs) to 84750 ng/mL (cholesterol esters), and example standard curves for LPE, SM, and PG, PI, PE, and LPC in (-ve ESI MS) are provided in Figure S10. Examination of the internal standard response from the pooled study QC data (see Table 3) indicated that the method provided acceptable accuracy and reproducibility with the observed CVs ranging from less than 3% for the PG­(15:0/18:1) (d7) in -ve ESI to *ca.* 17% for Cer­(d18:1/18:0) (d7) in +ve ESI. For all of the lipid standards in the study reference, blank matrix, and study QCs, the variation observed was less than 15% across all concentrations except for Cer­(d18:1/18:0) (d7) in the study reference QC, where it was less than 20% (Table 3). Together with other specific polar lipids, a total of 433 analytes were monitored (see ref 8) by the assay. These included 203 lipids only detected in these samples in +ve ESI, 194 detected only in -ve ESI, and a further 38 detected by both modes of ionization (Figure S11). The quantitative data obtained in this study are provided in Table S5. Multivariate statistical analysis of the quantitative lipid analysis data was initially performed using unsupervised PCA (Pareto scaling). Analysis of quantitative +ve ESI MS lipidomic data in this way showed that the blank matrix and gefitinib-dosed mouse liver extracts were clearly separated, with PCs 1 and 2 accounting for 44.2% and 10.6% of the variation, respectively. The study reference, blank matrix, and study reference QCs also clustered tightly together at the center of their respective groups, as illustrated in Figure S12.

**Table 3:** Comparison of the %CVs of the Internal Standard (IS) Response for Both +ve and -ve ESI using the Omics Lipid Screening Internal Standard^a^ ^,^ ^b^

		% CV blank matrix QC	% CV study ref QC	% CV study QC
**+ve ESI**	Gefitinib (d6)	8.33	7.35	5.80
	PC(15:0/18:1)(d7)	6.57	10.82	14.87
	LPC(18:1)(d7)	4.40	5.35	4.61
	LPE(18:1)(d7)	7.95	7.86	6.89
	MG(18:1)(d7)	4.38	7.24	8.33
	SM(d18:1/18:1)(d7)	2.03	2.78	1.97
	Cer(d18:1/16:0) (d7)	5.65	6.60	7.21
	Cer(d18:1/18:0) (d7)	4.19	16.99	4.53
	Cer(d18:1/24:0) (d7)	10.45	7.86	14.40
**-ve ESI**	PC(15:0/18:1)(d7)	11.99	12.45	11.14
	PE(15:0/18:1)(d7)	10.54	13.40	14.14
	PG(15:0/18:1)(d7)	2.74	2.03	2.59
	LPC(18:1)(d7)	3.14	4.01	2.93
	LPE(18:1)(d7)	7.53	10.20	9.21

a SIL internal standard concentrations are given in Table S1.

b Responses in QC Samples across the Run (*n* = 12).

PLS-DA of the targeted +ve HILIC-MS/MS data showed a clear separation of the sampling time points with PC1 and PC2 describing 18.2 and 17.1% of the variance in the data, respectively (Figure 3A,B). As seen for the +ve ESI model obtained for the untargeted analysis, the +ve ESI HILIC analysis also achieved high cross-validation (*R* ^2^ *Y* ≈ 0.95–1.00, *Q* ^2^ ≈ 0.90, CV accuracy ≈ 0.90) using 5-fold cross-validation (5-fold CV). The small *R* ^2^–*Q* ^2^ gap (<0.1) suggested that there was limited overfitting. Further, the 1,000-iteration permutation test yielded *p* < 0.001 (0/1000), showing that its performance exceeded that expected under random class assignment (Figure S13). As noted for the untargeted analysis, there was an observable time-related progression of the samples from the 0.5 h samples through the 1, 3, 8, and 24 h post-dose samples (Figure 3A). Analysis of the loadings data showed that the lipid features contributing most to the observed variation were PCs, SMs, TGs, and LPCs, with the lipids PC(34:1), PC(32:1), SM­(d40:1), and TG(50:1) the most significantly affected (Figure 3B). The resulting top 25 lipids from the VIP projection of the PLS-DA analysis of the +ve ESI data are provided in Table 4, with lipids in bold font representing those which were also identified as being important (based on VIP score) in both discovery and targeted methods.

**Figure 3:**
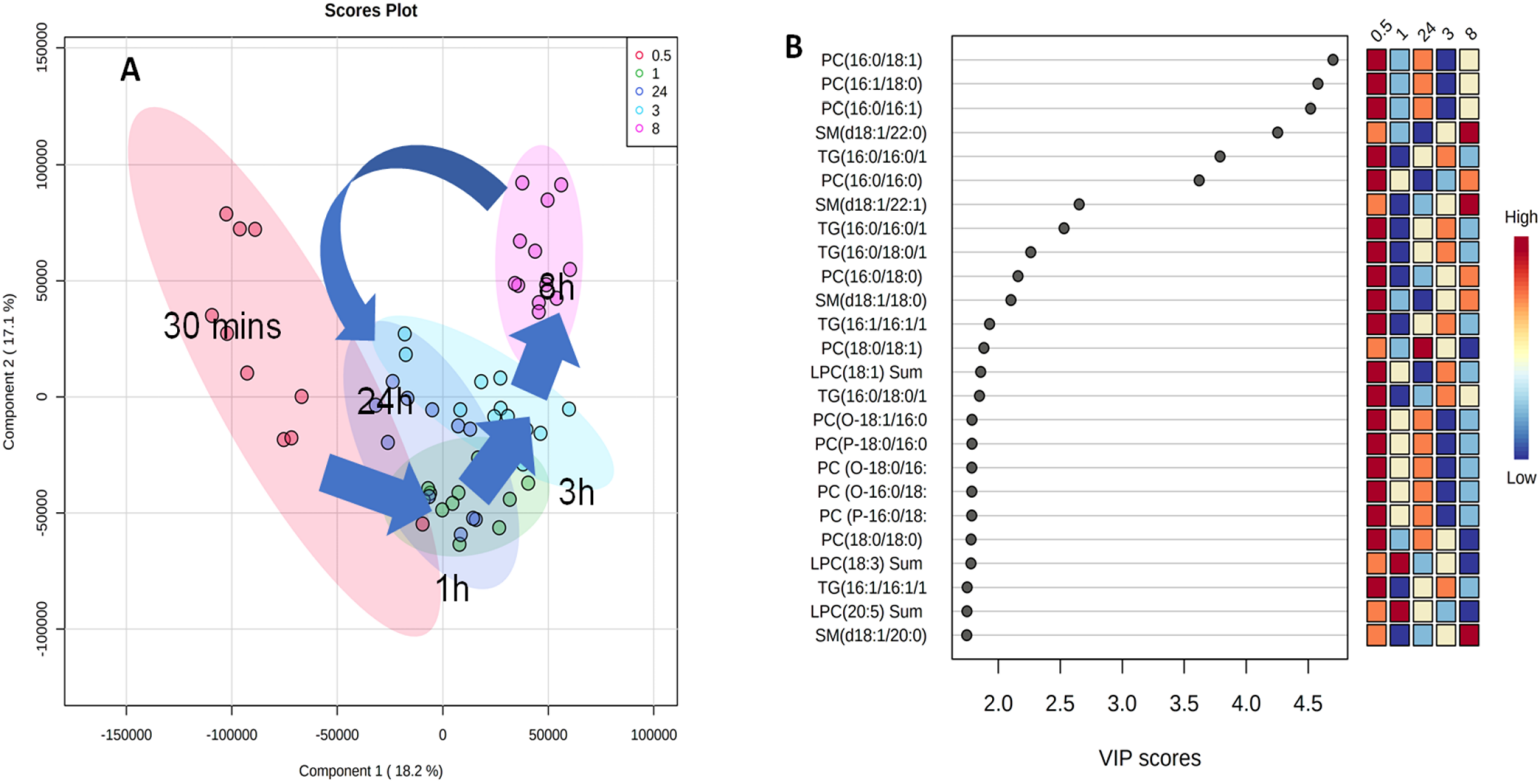
PLS-DA model of +ve ESI data HILIC LC-MS/MS (0.5–24 h post dose) excluding drug-related features and variables in less than 50% of the sample groups. Interquartile range filtering (IQR) and Pareto scaling applied; (A) scores plot and (B) top 25 analytes by variable importance in projection (VIP) (see also Figure S13).

**Table 4:** Top 25 Variables Importance in Projection (VIP) for +ve and -ve ESI^a^ from the Targeted LipidQuan Assay^b^ ^,^ ^c^

	lipidquan VIP lipids			
	positive ESI		negative ESI	
	library annotation	sum comp ID	library annotation	sum comp ID
1	**PC(16:0_18:1)**	**PC(34:1)**	FA(22:6)	FA(22:6)
2	**PC(16:1_18:0)** ^b^	**PC(34:1)**	**FA(16:0)**	**FA(16:0)**
3	**PC(16:0_16:1)** ^b^	**PC(32:1)**	FA(20:4)	FA(20:4)
4	SM(d18:1_22:0)	SM(d40:1)	FA(20:0)	FA(20:0)
5	**TG(16:0_16:0_18:1)** ^b^	**TG(50:1)**	**PG(16:0_18:1)**	**PG(34:1)**
6	PC(16:0_16:0)	**PC(32:0)**	FA(16:1)	FA(16:1)
7	SM(d18:1_22:1)	SM(d40:2)	FA(18:1)	FA(18:1)
8	**TG(16:0_16:0_16:1)**	**TG(48:1)**	LPE(18:0)	LPE(18:0)
9	**TG(16:0_18:1_18:1)**	**TG(52:1)**	FA(18:0)	FA(18:0)
10	**PC(16:0_18:0)**	**PC(34:0)**	**PE(18:0_20:4)**	**PE(38:4)**
11	SM(d18:1_18:0)	SM(d36:1)	FA(22:0)	FA(22:0)
12	**TG(16:1_16:1_18:0)** ^b^	**TG(50:2)**	FA(22:5)	FA(22:5)
13	PC(18:0_18:1)	PC(36:1)	PG(18:2_18:2)	PG(36:4)
14	LPC(18:1)	LPC(18:1)	LPE(18:1)	LPE(18:1)
15	**TG(16:0_18:0_18:2)**	**TG(52:2)**	FA(18:2)	FA(18:2)
16	**PC(O-18:1_16:0)**	**PC(O-34:1)**	FA(22:4)	FA(22:4)
17	**PC(P-18:0_16:0)**	**PC(P-34:0)**	**LPE(16:0)**	**LPE(16:0)**
18	**PC(O-18:0_16:0)**	**PC(O-34:0)**	**PG(16:0_18:2)**	**PG(34:2)**
19	**PC(O-16:0_18:1)**	**PC(O-34:1)**	**PC(16:0_20:4)**	**PC(36:4)**
20	**PC(O-16:0_18:0)**	**PC(O-34:0)**	LPC(20:4)	LPC(20:4)
21	PC(18:0_18:0)	PC(36:0)	FA(24:5)	FA(24:5)
22	LPC(18:3)	LPC(18:3)	**PE(18:1_20:4)** ^b^	PE(38:5)
23	TG(16:1_16:1_18:1)^b^	TG(50:3)	**PC(16:0_18:2)** ^b^	**PC(34:2)**
24	LPC(20:5)	LPC(20:5)	PG(16:0_16:1)	PG(32:1)
25	SM(d18:1_20:0)	SM(d38:1)	PE(18:1_18:2)	PE(36:2)

a Excluding gefitinib/metabolites.

b Lipids that were also significant in the untargeted discovery method (see Table S3). C16 and C16-containing lipids are indicated in **bold**.

c PC-O- and PC-P-containing lipids were differentiated based on specific MRM transitions.^8^

MVA showed that, *e.g*., LPC(18:1) was significantly elevated in liver samples from gefitinib-treated animals compared to the blank matrix, whereas SM(36:3), GlcCer(34:1), and CAR(18:2) (linoleoyl-L-carnitine) were significantly lower in relative abundance, Figure S14. PCA and PLS-DA of the -ve ESI HILIC-MS/MS data set showed a similar pattern to that obtained from the +ve ESI data, with the treated group samples clearly separated from the blank matrix and study reference QCs, and with the latter clustering tightly together within their respective groups (see Figure S15). PC1 and PC2 accounted for 47.5 and 17.5% of the variance in the PCA scores plot, while for PLS-DA, PC1 and PC2 accounted for 47.3 and 12.2 of the observed variation in these data, respectively. PLS-DA of the liver extracts of the gefitinib-dosed mice showed a clear anticlockwise time-based trajectory from the 0.5 h to 1, 3, 8, and 24 h samples (Figure 4), with PC1 and PC2 describing 28.2% and 23.1% of the variation in these data, respectively. The PLS-DA model derived from these data provided good cross-validation performance (*R* ^2^ *Y* ≈ 0.95–0.98, *Q* ^2^ ≈ 0.95–0.98, CV accuracy ≈ 0.85–0.88) with limited overfitting (the *R* ^2^–*Q* ^2^ gap was <0.1) using 5-fold cross-validation (5-fold CV). A 1000-iteration permutation test yielded *p* < 0.001 (0/1000), confirming the model performance substantially exceeded the result expected under random class assignment.

**Figure 4:**
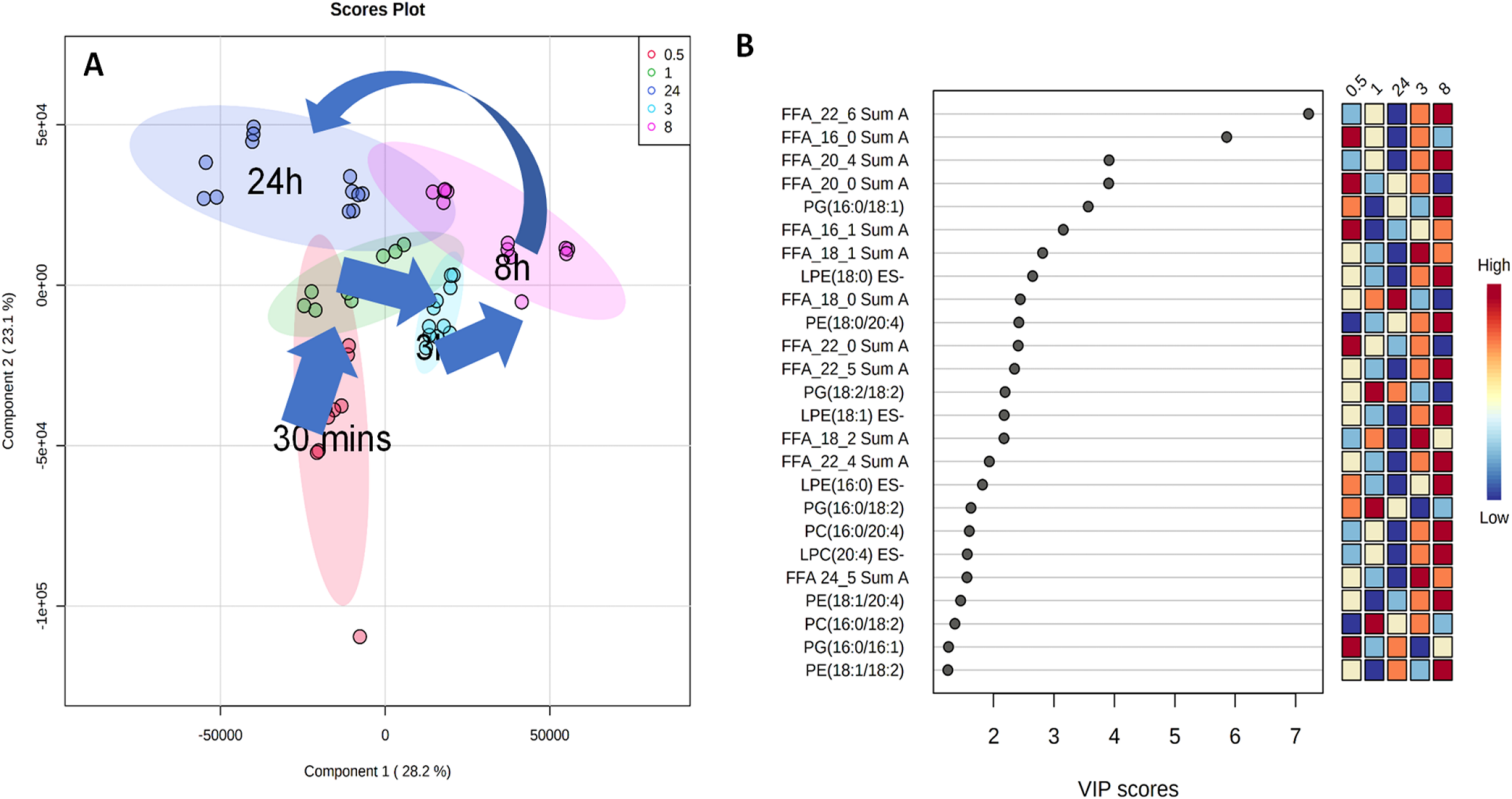
PLS-DA of -ve ESI HILIC-MS/MS targeted gefitinib IV dose group liver data using Pareto scaling; (A) score plot and (B) the top 25 lipids by variable importance in projection (VIP). FFA Sum A = free fatty acids sum composition.

The lipids contributing significantly to the time point separation included free FAs, PGs, PCs, LPEs, and PEs. The resulting top 25 lipids from the PLS-DA (VIP analyses) are given in Table 4, for both + and -ve ESI. As this table shows, some of these lipids contributing significantly to the observed variance in these data were also identified in the untargeted discovery analyses and are highlighted with a bold font. One of the most interesting features of the lipids listed in Table 4 is the preponderance of those containing the C16:0 fatty acid (FA) palmitate that populate it. These C16:0-containing lipids comprised 21 of the 50 detected as being statistically most altered. Given the importance of palmitate in mammalian biochemistry, this is perhaps not too surprising, and while diet is a major source, *de novo* lipogenesis (DNL) is also important.^19^ The DNL of palmitate proceeds via acetyl-CoA and uses acetyl-CoA-carboxylase to convert it to malonyl-CoA for subsequent addition of 2-carbon subunits catalyzed by fatty acid synthase (FASN) until chain elongation to C16 is achieved. From thereon, palmitate can be converted to other lipids, including C16:1 (palmitoleic acid) by desaturation, or further modified by chain elongation to *e.g*., C18:0 (stearic acid) and above. All of these FFA are then available for incorporation into other complex lipids resulting in, this instance, the pattern illustrated in Figure 5.

**Figure 5:**
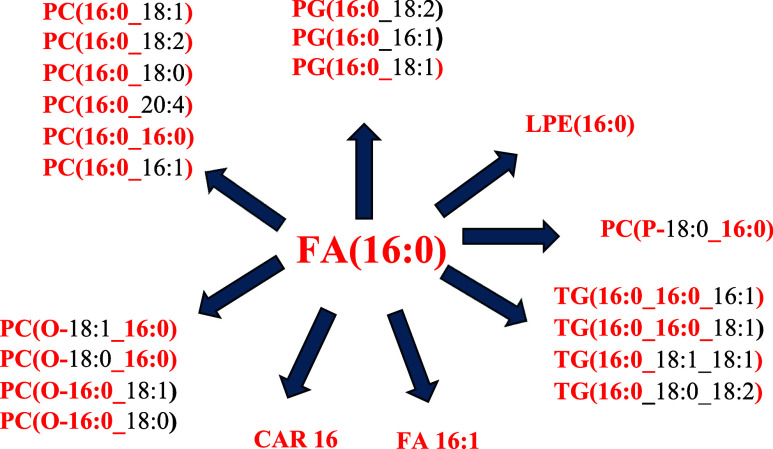
Statistically significant lipids derived from C16:0 in the top 50 lipids that were shown to be changed in liver extracts using the HILIC-based targeted assay.

### Potential Gefitinib–Lipid Correlations

Previously, an apparent correlation between changes in endogenous polar metabolite concentrations in urine and systemic gefitinib/metabolite concentrations obtained for samples from this study has been identified.^3^ More recently, changes in the profiles of certain plasma lipids were found to be well correlated with systemic drug and drug metabolite concentrations.^4^ In the case of potential effects on liver lipid changes in 5 PCs (PC(32:1), PC(34:0), PC(34:1), PC(34:2), PC(36:1)) and 2 TGs (TG(50:1), TG(50:3)) were seen in the quantitative +ve ESI MS data as shown in Figure 6. The PCs generally showed an increase in abundance from the 0.5 h post-dose sample to reach a maximum observed value at the 3 h time point (although some appeared to undergo an initial reduction from the 0.5 h time point) before gradually declining again by the 8 and 24 h time points. PC(34:1) and PC(34:2) showed the largest potential response to gefitinib administration with an almost 4-fold increase in abundance from the 0.5 h value by 3 h before returning to the initial abundance by 8 h. However, the abundance of the 2 TGs initially fell from their 0.5 h post dose values to a minimum observed value at 1 h, before rising to again at 3 h, before a further decline by 8 h post dose (Figure 6).

**Figure 6:**
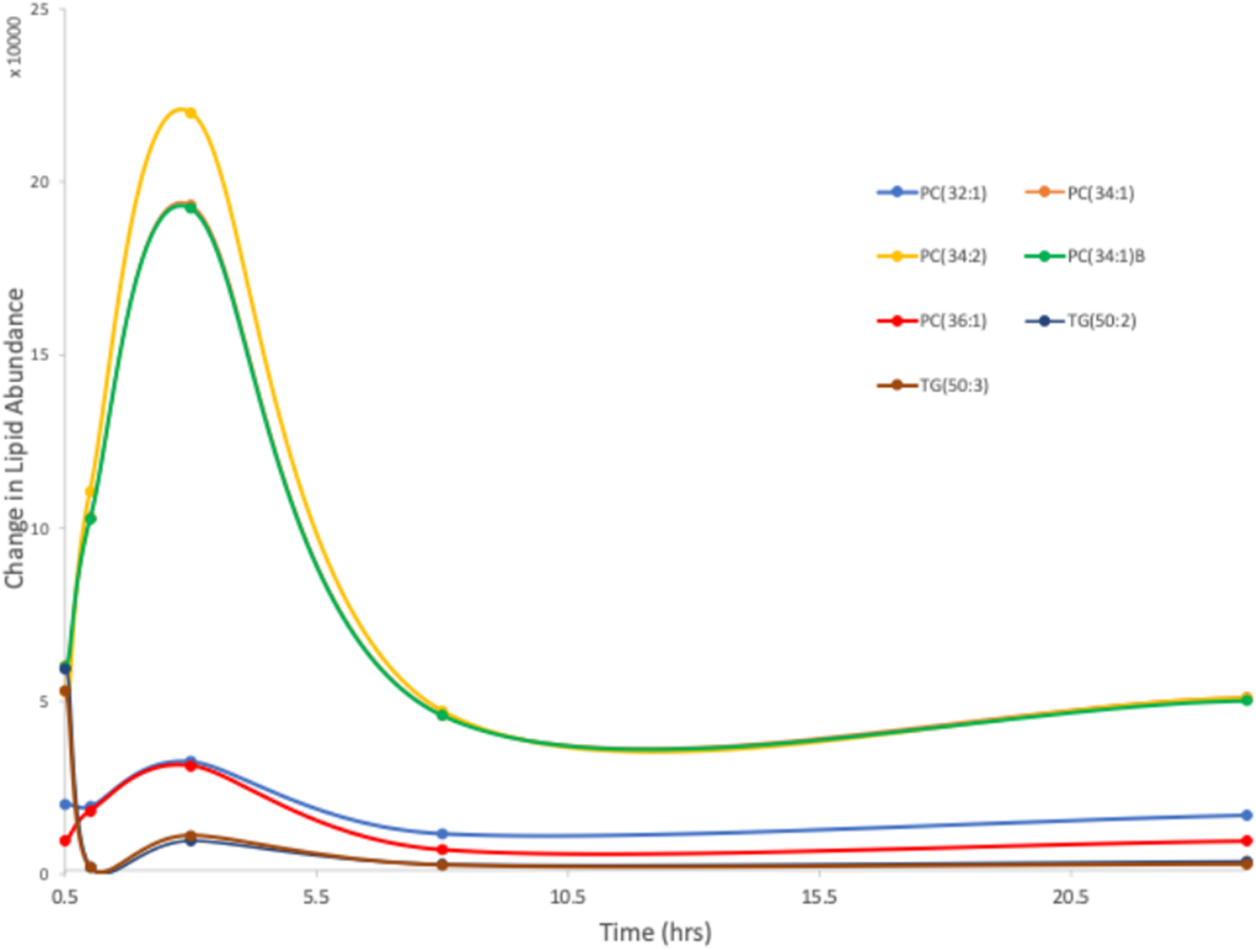
Comparison of changes in lipid abundance following intravenous administration of gefitinib at 10 mg/kg to male mice. +ve ESI data for PC(32:1), PC PC(34:0), PC PC(34:1), PC(34:2), PC(36:1), TG(50:1), TG(50:3).

A similar analysis for the targeted -ve ESI HILIC-MS/MS data showed that the liver tissue concentrations of, *e.g*., PC(36:4) and PE(38:4), were also affected by drug administration, with concentrations increasing to a maximum at 3 h before again declining to trough levels at 8 and 24 h (Figure S16).

Interestingly, the relative abundances of the PEs were also observed to reduce in the plasma samples from this study,^4^ whereas the PCs and LPEs exhibited the opposite trend in changes in plasma compared to the changes in hepatic abundance following gefitinib dosing.

As noted above, peak observed concentrations of gefitinib in liver extracts of *ca.* 300 ng/mg were seen 0.5 h after dosing reducing to 250 ng/mg by 1 h remain at that level until 3 h post dose, decreasing from this time onward to below the limit of quantification for the 24 h samples (Figure 2A) (gefitinib tissue *vs* plasma concentrations are shown in Figure 2B). As noted above, previously we have seen that changes in some plasma lipid concentrations were strongly correlated with those of gefitinib/gefitinib metabolites^4^ with some plasma lipid abundances (*e.g*., LPCs and PIs) apparently increased with high gefitinib concentrations, while others (*e.g*., PCs and TGs) decreased. However, when liver lipid abundances were compared with drug concentrations, a more complex picture was obtained, as shown in Figure S17.

### Targeted Analysis of Free Fatty Acids (FFA)

As can be seen from the heatmap in Figure 4, significant effects on FFAs such as FA16:0, 16:1, 18:0, 18:1, 18:2, 20:4, 22:5 and 22:6 were seen following gefitinib administration when liver extracts were analyzed using -ve ESI HILIC-MS/MS (see Figure S18A,B). Thus, FA(16:0), FA(16:1), FA(18:0), FA(18:1) and FA(20:4) appeared to decline from their 0.5 h post dose abundances to a minimum abundance in the 1 h liver extracts dose before increasing again in the 3–8 h extracts and then falling once more by 24 h post dose (Figure S18A). In contrast to these FFAs, others such as FFAs (18:2) and (22:6) rose from their observed abundances at 0.5 h post drug administration, slowly increasing in quantity to 8 h before a gradual decline by 24 h. Others, such as FFA(22:5), seemed to be relatively stable (Figure S18B) until the end of the study. In Figure S19, the concentrations of gefitinib in the plasma are plotted against FFA abundances in the liver extracts and show, as did the PC and TG described above, a complex pattern, with no obvious correlation between drug concentration and the liver extracts.

### Targeted Analysis of Liver Acyl Carnitines (CAR)

Due to their favorable retention characteristics in the HILIC-based chromatographic system used for targeted analysis, the qualitative determination of several acyl carnitines (CAR) was possible (in +ve ESI) in addition to the lipids and FFA in these extracts (see Figure S20). While not obtained using a validated method, the peak area and t_R_ reproducibilities seen for the acyl carnitines in the study QC samples were acceptable (see Figure S21), thus providing a further window into the potential impact of drug administration on the liver.

The short-chain acyl carnitines showed a variety of responses to gefitinib administration. So, while carnitine itself and CAR 2:0 showed no distinct trend, the abundance of CAR 4:0 appeared to reduce from 0.5 h post dose to a minimum value 8 h after drug administration before increasing again at 24 h post dose. CAR 8:0 appeared to show an initial reduction in relative abundance from 0.5 h, reaching a minimum value in the 1 h samples, before increasing in the 8 h extract and then falling back by 24 h post dose (Figure S22). This pattern was replicated by the longer chain CAR 14:0, 18:0, and 18:1 carnitines (Figure S23), but, in contrast, CAR 16:0 showed a marked reduction in abundance from 0.5 h post dose, only recovering slightly in the 24 h liver extracts. Clearly, if these changes are drug-related, the effects on these important intermediates in fatty acid metabolism (which facilitate the transport of fatty acids into mitochondria) may have important consequences for *e.g*., energy production. Also, it may be noteworthy that the CAR 14:0, CAR 16:0, CAR 18:0, and CAR 18:1 have been shown to have other effects, including, interestingly, the ability to cause the activation of pathways involved in stress, cellular inflammation, and cell death in a model system.^20^ In this study, the authors noted that “*Differentiated C*_2_ *C*_12_ *myotubes treated with* l *-C14, C16, C18, and C18:1 carnitine displayed dose-dependent increases in IL-6 production with a concomitant rise in markers of cell permeability and death, which was not observed for shorter chain lengths*”.^20^ It may be no coincidence therefore that these are the same acyl carnitines that appeared to be affected by gefitinib administration and may reflect, at least in part, aspects of the drug’s MOA.

As indicated in the introduction, this lipidomic study was undertaken on samples derived from a study to determine the pharmacokinetics and metabolic fate of gefitinib in the mouse. While it was not a primary aim of that work, the opportunity afforded by having liver samples has been taken to examine the value of combining both untargeted and targeted methods of lipid analysis as a means of increasing both the breadth and depth of lipidome coverage. In this respect, it is noteworthy that, as would perhaps be expected based on the differences in chromatographic selectivity between the lipophilicity-based reversed-phase and class-based HILIC separations, there was little overlap in their most statistically significant lipid lists. To this end, the targeted HILIC methodology employed here was specifically designed to overcome the early coelution of the most polar lipids (LPCs/LPEs) seen with the RP-UHPLC-IM-MS method and facilitate the analysis of bioactive lipids such as PC, PI, SM, LPE, and LPCs. A comparison of the PLS-DA analysis of both untargeted and targeted data sets showed that of the 25 lipids identified in the VIP list for the +ve ESI MS data, 8 were detected by both approaches, with 3 also present in both the -ve ESI MS VIP lists (Table 4). Both modes of analysis also appeared to show time-related trajectories in their data, albeit more complex and difficult to interpret than the changes previously noted for lipids in the systemic circulation after both IV and PO administration.^4^ So, following PO dosing, plasma PCs, LPCs, PEs, phosphatidylinositol (PI), phosphatidylserines (PS), and TGs all appeared to be dysregulated.^4^ In the case of the IV dose, effects on PE(36:2), PE(36:3), PE(38:3), PE(40:2), PE(40:6), PC(34:2), PC(38:6), and TGs(56:8) and (60:12) were seen.^4^ The responses of the affected plasma lipids to the drug included both increases and decreases in relative abundance, but with a general return toward predose values by 24 h post dose.

In the present study of the liver response to gefitinib, notwithstanding the limited number of samples and sampling times available for analysis, both untargeted and targeted lipidomics may have detected drug-modulated effects on the liver lipidome. The untargeted lipidomics analysis showed an apparent reduction in hepatic PC and PE concentrations and a concomitant increase in the amounts of the LPCs following exposure to gefitinib. This reduction in PCs and the corresponding increase in LPCs are consistent with known lipid biochemistry, as LPCs, as noted above, are formed from PCs by phospholipase A2 in the Lands Cycle.^21^ Similar effects on the liver lipid profiles have been detected previously following drug administration, *e.g.*, the exposure of rats to the multiresistance tuberculosis drug ethionamide,^22^ where reductions in hepatic PCs, LPCs, LPEs, PEs, PIs, and SMs and an increase in TGs were noted (with an inverse relationship between plasma and hepatic TG concentrations). It is thus tempting to attribute these changes in lipids, such as the increases observed in the abundance of the PCs, the secondary maximum of the TGs, and the peak concentration for the LPCs/LPEs, etc., to the action of gefitinib. While it is possible these effects may indeed be due to gefitinib (which appears to show a secondary maximum at 3 h, Figure 2), they may also have been due, at least in part, to metabolites such as M6 (M537194), rather than the drug itself. The targeted method detected complex time-related changes in the livers of gefitinib-dosed mice, with classes such as the PCs, LPCs, PEs, PIs, FFAs, and TGs being most affected.^4^ TG concentrations showed an immediate fall from their maximum at the 0.5 h time point and remained low until the end of the study. In contrast, while FFA concentrations initially fell following dosing, they had risen again by 3 h, staying high until the 8 h time point before falling again by 24 h post dose. The acyl carnitines showed a general decline from their 0.5 h maxima with a subsequent rise at 8/24 h. These data perhaps suggest that liver TG, FFA, and acyl carnitine abundances were modulated by gefitinib administration and liver drug tissue concentration. An obvious conjecture is that, as the TG represents a storage form of the FA, their release via lipolysis provided FFAs which were converted into acyl-CoA esters and thence into acyl carnitines for transport into mitochondria and subsequent β-oxidation and ATP production. However, as noted above, the long-chain acyl carnitines also have effects on pathways involved in stress, cellular inflammation, and cell death in model systems.^20^ In terms of gefitinib’s MOA, it is interesting to note that lipid homeostasis in human cells is known to be regulated via sterol regulatory element-binding proteins (SREBPs), which equilibrate intracellular levels of FAs and cholesterol.^23^ FASN, as noted above, is important in the synthesis of palmitate and is upregulated in several cancers,^24–26^ and EGFR has been shown to mediate TKI resistance via FASN.^27^ As was clear from the results of this study, both palmitate and many of the lipids into which it is incorporated were among those that were most significantly affected in these gefitinib-treated mice.

While acknowledging the limitations of this investigation, resulting particularly from the small number of mice involved, the apparent widespread effects of gefitinib on the liver lipidome, and the complex behaviors seen here, suggest that a more detailed study might be warranted. As the intended targets for these drugs are tumors, looking for these effects, e.g., in *in vitro* tumor models, would seem to represent an obvious way forward. The changes observed in this study may reflect both the drugs’ MOA and secondary, “off-target”, pharmacology. In addition, it is also possible that at least some of these effects are due to the accumulation of metabolites such as M6 (M537194) (or other undetected metabolites), which had peak observed concentrations in the 3 h liver extract rather than gefitinib itself. However, making definitive conclusions that the effects seen here are entirely attributable to gefitinib and its metabolites based on the results of this preliminary study is clearly not possible. For example, some of the effects on the acyl carnitines or PC/LPC ratios seen here could also have been associated with, *e.g.*, stress^20,28^ or inflammatory responses,^29^ rather than drug treatment *per se*.

Should these *in vivo* effects be replicated and confirmed using, e.g., carefully designed flux experiments *in vitro*, then a more detailed understanding of the effects of gefitinib (and selected metabolites) should be possible. If these disturbances in lipid metabolism do reflect drug efficacy, then it might be worth pursuing the changes previously observed in plasma lipid profiles as a relatively noninvasive means, compared to tumor biopsies, of monitoring the efficacy of gefitinib (and similar drugs) in patients undergoing therapy.

## Conclusions

The combination of both untargeted and targeted lipidomic phenotyping for profiling the same samples provided a more comprehensive assessment of changes in the liver lipidome of mice. Untargeted lipid analysis by RP-UHPLC-IM-MS revealed reductions in the PCs and PEs and rises in the amounts of LPCs, which may have occurred as a result of the IV administration of gefitinib. Targeted analysis using HILIC-MS showed apparent time-related effects on the profiles of PCs, SMs, TGs, LPCs, FFAs, PGs, LPEs, PEs, and acyl carnitines. Notwithstanding the limitations of the study in terms of the small numbers of animals sampled, these measurements appeared to reveal complex temporal drug-related (pharmacolipidodynamic) effects by the EGFR inhibitor gefitinib on liver lipid profiles. If confirmed with further investigations, these may provide a more comprehensive view of the effects of TKI inhibition and reveal more about the mode of action of these important drugs.

## Supplementary Material







## Data Availability

The MS lipidomic data obtained here are available from the Metabolights data repository (EMBL-EBI, Wellcome Genome Campus, Cambridgeshire, UK) with the data set identifier REQ20260112216019.

## Notes

Stable isotope-labeled lipid standards in SPLASH LIPIDOMIX, Ceramide LIPIDOMIX (Table S1A); Avanti odd-chain LIPIDOMIX and gefitinib calibration curve concentrations (Table S1B); the top 25 lipids in the +ve and -ve ESI MS detected using discovery LC-IM-MS (Table S3); concentrations of gefitinib in liver tissue extracts (Table S4); mass chromatograms for RP-UPLC-HDMS^e^ liver extracts in +ve and -ve ESI (Figure S1); PCA and PLS-DA +ve ESI HDMS^e^ data (Figure S2); profiles of gefitinib and its M6 and M3 metabolites in liver lipid extracts (Figure S3); PCA and PLS-DA of RP-UPLC-IM-MS +ve ESI data (0.5–24 h post dose) data excluding drug-related features with cross-validation and permutation tests (Figure S4); PCA and PLS-DA of RP-UHPLC-IM-MS -ve ESI data (0.5–24 h post dose) excluding drug-related features with cross-validation and permutation tests (Figure S5); PLS-DA of -ve ESI RP-UHPLC-IM-MS data (Figure S6); trend lines for LPCs (20:4), (18:2), (34:1), and (36:4) PCs in +ve ESI (Figure S7); responses for gefitinib and gefitinib d6 in all study samples and QCs and examples of gefitinib and gefitinib-d6 in a QC (Figure S8); mass chromatograms for HILIC-MS of liver lipid extracts in -ve and +ve ESI (Figure S9); calibration lines for PG, PI, PE, LPC (-ve ESI) and SM, LPE and gefitinib (+ve ESI-) (Figure S10); Venn diagram of lipids monitored by HILIC-MS (+ve and -ve ESI) showing overlap (Figure S11); PCA score plot for targeted +ve ESI HILIC-S data (Figure S12); PCA and PLS-DA of +ve ESI HILIC-MS data (0.5–24 h post dose) excluding drug-related features (Figure S13); *t*-test +ve ESI MS data, treated *vs* matrix control (Figure S14); PCA and PLS-DA of -ve ESI HILIC-MS data (Figure S15); changes in abundance PC(36:4) and PE(38:4) in the -ve ESI HILIC-MS data (Figure S16); changes in abundance on selected PC and TG *vs* time (Figure S17). changes in abundance of selected liver FFA *vs* time (Figure S18); FFA abundances in liver extracts *vs* plasma concentrations of gefitinib (Figure S19); acyl carnitine *t*_R_ and peak areas across the liver sample analysis (Figure S20); separation of acyl carnitines in extracts with detection by +ve ESI MS (Figure S21); relative intensity changes in short-chain acyl carnitines (Figure S22); and relative intensity changes in long-chain acyl carnitines (Figure S23) (PDF)
Sample run list for both untargeted and targeted lipidomic analysis (Table S2) (XLSX)
Quantitative lipidomic data for liver extracts (Table S5) (XLSX)
