# Supplementary material for: Maximizing Lipidome Coverage of Mouse Liver Following the IV Administration of Gefitinib by Combining Both UHPLC-MS-Based Untargeted and Targeted Lipidomics
Source: J Proteome Res. 2026 Jun 13;25(7):3521–32. doi: 10.1021/acs.jproteome.5c01271 (PMC13339825; doi:10.1021/acs.jproteome.5c01271)
Supplement: Supplementary file 1 [file pr5c01271_si_001.pdf]

SUPPLEMENTARY INFORMATION

Maximizing Lipidome Coverage of Mouse Liver Following the IV Administration of Gefitinib by Combining both UHPLC-MS-based Untargeted and Targeted Lipidomics

Robert S Plumb[1,4], Nyasha Munjoma[2], Lee A Gethings[2], Ian D Wilson[3,4*]

[1]Waters Corporation, 34 Maple St, Milford MA, 01757, USA

[2]Waters Corporation, Stamford Ave, Wilmslow, SK9 4AX, UK

[3]Division of Systems Medicine, Department of Metabolism Department of metabolism, Digestion and Reproduction, Imperial College, Burlington Danes Building, Du Cane Road, London W12 0NN, UK.

[4]Biochemistry, Cell and Systems Biology, Faculty of Health and Life Sciences, University of Liverpool, Brownlow Hill, Liverpool, L69 3GB

*Corresponding author: Email: i.wilson@imperial.ac.uk

Contents:

Table S1A: Stable isotope labelled lipid standards in SPLASH LIPIDOMIX™, deuterated Ceramide LIPIDOMIX™ and deuterated gefitinib used as IS's

Table S1B: Avanti Odd-Chain LIPIDOMIX™ & Gefitinib calibration curve concentrations

For **Table (xlsx) S2** Sample Run List for Both Untargeted and Targeted Lipidomic Analysis Supplementary Excel spreadsheet

Table S3. The top 25 lipids in the +ve and -ve ESI MS variable importance in projection (VIP) PLS-DA models detected using the discovery LC-IM-MS methodology.

Table S4: Concentrations of gefitinib in tissue extracts and back calculated liver tissue following IV administration at 10 mg/Kg to male mice (3 sig fig)

Table S5 (xlsx): Quantitative lipidomic data for liver extracts

Figure S1: Reversed-phase UHPLC-HDMS^e mass chromatograms of mouse liver extracts with (A) +ve ESI and (B) -ve ESI obtained using the discovery LC-IM-MS methodology. .

Figure S2: PCA(A) and PLS-DA (B) multivariate statistical model of +ve ESI HDMS^e data obtained using the discovery LC-IM-MS methodology, excluding variables present in less than 50% of the pooled study QC samples (standards, gefitinib and metabolites included). Interquartile Range Filter (IQR) Pareto Scaling applied. (treated (blue), matrix control (non-treated (red)) and study ref (green).

Figure S3. Profiles of gefitinib and its associated metabolites M6 and M3 in the liver lipid extracts following IV administration of the drug at 10 mg/Kg to male mice (+ve ESI-MS).

Figure S4. PCA and PLS-DA of RP-UHPLC-IM-MS +ve ESI data (0.5-24 h post dose) data obtained excluding drug-related features and variables in less than 50% of the sample groups. Interquartile range filtering (IQR) and Pareto scaling was applied to generate these models. 5‑fold cross‑validation (CV 5‑fold) of the supervised model showed $R^2 \approx 0.98–1.00$, $Q^2 \approx 0.90–0.92$ and Accuracy ≈ 0.88–0.90. Using 5 components achieved high cross validated performance ($R^2Y \approx 0.95–1.00$, $Q^2 \approx 0.90$, CV accuracy ≈ 0.90). The R^2–Q^2 gap was small (<0.1), suggesting limited overfitting. A 1,000 iteration permutation test yielded $p < 0.001$ (0/1000), confirming model performance exceeded that expected under random class assignment.

Figure S5. PCA and PLS-DA of RP-UHPLC-IM-MS -ve ESI data (0.5-24 h post dose) excluding drug-related features and variables in less than 50% of the sample groups. Interquartile range filtering (IQR) and Pareto scaling was applied to generate these models. CV 5-fold testing achieved high cross-validated performance with 5 components ($R^2Y \approx 0.98$, $Q^2 \approx 0.90$, CV accuracy ≈ 0.90). The R^2–Q^2 gap was small (<0.1), indicating limited overfitting. A 1000-iteration permutation test showed $p < 0.001$ (0/1000), confirming that model performance exceeded that expected by chance.
Figure S6: The PLS-DA of -ve ESI RP-UHPLC-IM-MS Data shown in Figure S5 A) Score plot and (B) top 25 variable importance in projection (VIP).
Figure S7: Trend lines for LPCs (20:4) and (18:2) and (34:1) and (36:4) PCs in +ve ESI -MS.
Figure S8: The upper trace (A) shows the responses for gefitinib (grey) and gefitinib d6 (pink) in all of the study samples and QCs whilst the lower trace (B) shows a typical example for gefitinib (grey) gefitinib-d6 (pink) in a typical study QC.
Figure S9. HILIC-based separation of liver lipid extracts using a 2.1 x 100mm ACQUITY BEH Amide 1.7µm column, eluted with a 95% acetonitrile 5% 10 mM ammonium acetate (v/v) Vs 50% ACN, 50% water, 10 mM ammonium acetate (v/v) gradient at 0.6 mL/min over 5 min with -ve (**A**) and +ve ESI MS detection (**B**).
Figure S10: Calibration lines for PG, PI, PE, LPC (-ve ESI MS) and SM, LPE and gefitinib (+ve ESI-MS)
Figure S11. Numbers of lipids monitored by the targeted HILIC-MS method in liver extracts in +ve and -ve ESI showing overlap in those detected using both methods.
Figure S12: PCA score plot for targeted +ve ESI MS data. Variables in less than 50% of samples excludes, standards, gefitinib and metabolites included. Interquartile range filtering (IQR) and Pareto scaling was applied
Figure S13. PCA and PLS-DA of +ve ESI HILIC LC-MS/MS +ve ESI data (0.5-24 h post dose) excluding drug-related features and variables in less than 50% of the sample groups. Interquartile range filtering (IQR) and Pareto scaling was applied to generate these models. 5-fold cross-validation (CV 5-fold) achieved high cross-validated performance ($R^2Y \approx 0.95$–1.00, $Q^2 \approx 0.90$, CV accuracy ≈ 0.90). The R^2–Q^2 gap was small (<0.1), suggesting limited overfitting. A 1,000-iteration permutation test yielded $p < 0.001$ (0/1000), confirming model performance exceeded that expected under random class assignment.
Figure S14: T-Test +ve ESI MS data, treated (green) vs matrix control (non-treated) (red). Peak intensity, excluding variables present in less than 50% of pooled study QC samples. Pareto Scaling applied (excludes standards but with gefitinib and its associated metabolites included).
Figure S15: PCA (A) and PLSDA (B) of -ve ESI-MS data. Peak intensity, excluding variables present in less than 50% of samples, Pareto scaling (standards, gefitinib and its associated metabolites included).
Figure S16: Comparison in changes of lipid abundance following intravenous administration of gefitinib at 10 mg/kg to male mice. -ve ESI PC(36:4), PE(38:4).
FigureS17: Impact of gefitinib tissue concentrations on selected PC and TG lipid abundances.
Figure S18: Changes in abundance of selected liver FFA vs time following the IV administration of gefitinib at 10 mg/Kg to mice.
Figure S19: Comparison of FFA abundances in liver extracts with plasma concentrations of gefitinib following IV administration at 10 mg/Kg to mice.
Figure S20: Acyl carnitine t_R (upper) and peak areas (lower) across the randomised sample analysis of liver extracts following IV dosing with gefitinib at 10 mg/Kg to the mouse.
Figure S21: Separation of acyl carnitines in mouse liver extracts with detection by +ve ESI MS following the administration of gefitinib at 10 mg/Kg to mice
Figure S22: Relative intensity changes for short chain acylcarnitines in mouse livers over time following IV dosing with gefitinib at 10 mg/Kg**.**
Figure S23: Relative intensity changes in long chain acylcarnitines in mouse livers over time following IV dosing with gefitinib at 10 mg/Kg.

Table S1A: Stable isotope labelled lipid standards in SPLASH LIPIDOMIX™, deuterated Ceramide LIPIDOMIX™ and deuterated gefitinib used as ISs in 3:1 (v/v) DCM/MeOH

Lipid class	Ionization mode	Mixture Component Target	Initial concentration (µg/mL)	Concentration in 3:1 (v/v) DCM/MeOH (µg/mL)	Concentration in 3:1 (v/v) DCM/MeOH (ng/mL)
Phosphatidylcholine (PC)	Both	PC(15:0/18:1)(d7)	160	**0.640**	**640**
Phosphatidylethanolamine (PE)	Negative	PE(15:0/18:1)(d7)	5	**0.020**	**20**
Phosphatidylserine (PS)	Negative	PS(15:0/18:1)(d7)	5	**0.020**	**20**
Phosphatidylglycerol (PG)	Negative	PG(15:0/18:1)(d7)	30	**0.120**	**120**
Phosphatidylinositol (PI)	Negative	PI(15:0/18:1)(d7)	10	**0.040**	**40**
Phosphatidic acid (PA)	Negative	PA(15:0/18:1)(d7)	7	**0.028**	**28**
Lysophosphatidylcholine (LPC)	Both	LPC(18:1)(d7)	25	**0.100**	**100**
Lysophosphatidylethanol-amine (LPE)	Both	LPE(18:1)(d7)	5	**0.020**	**20**
Cholesterol Ester (Chol Ester)	Positive	CE(18:1)(d7)	350	**1.400**	**1400**
Monoacylglycerols (MG)	Positive	MG(18:1)(d7)	2	**0.008**	**8**
Diacylglycerols (DG)	Positive	DG(15:0/18:1)(d7)	10	**0.040**	**40**
Triacylglycerols (TG)	Positive	TG(15:0/18:1/15:0)(d7)	55	**0.220**	**220**
Sphingomyelins (SM)	Positive	SM(d18:1/18:1)(d7)	30	**0.120**	**120**
Cholesterol (Chol)	Positive	Cholesterol (d7)	100	**0.400**	**400**
Ceramide (Cer)	Positive	Cer(d18:1/16:0) (d7)	21.8	**0.087**	**87**
Ceramide (Cer)	Positive	Cer(d18:1/18:0) (d7)	11.5	**0.046**	**46**
Ceramide (Cer)	Positive	Cer(d18:1/24:0) (d7)	26.3	**0.105**	**105**
Ceramide (Cer)	Positive	Cer(d18:1/24:1) (d7)	13.1	**0.052**	**52**
Drug	Positive	Gefitinib (d6)		**3.000**	**3000**

Table S1B: Avanti Odd-Chain LIPIDOMIX™ & Gefitinib calibration curve concentrations (ng/mL) prepared in 1:2 (v/v) IPA:ACN

Standard Concentrations (ng/mL)

	LPG(17:1)	LPA(17:1)	LPI(17:1)	LPS(17:1)	LPC(17:1)	LPE(17:1)	DG(17:0/17:0)	TG(17:0/17:0/17:0)	SM(d18:1/12:0)	PC(17:0/14:1)	PS(17:0/14:1)	PG(17:0/14:1)	PA(17:0/14:1)	PE(17:0/14:1)	PI (17:0/14:1)	CE(17:0)	Gefitinib
1	130	150	130	130	5750	120	3000	15000	6500	37750	1800	900	150	1200	2000	84750	1000
2	65	75	65	65	2875	60	1500	7500	3250	18875	900	450	75	600	1000	42375	750
3	52	60	52	52	2300	48	1200	6000	2600	15100	720	360	60	480	800	33900	600
4	26	30	26	26	1150	24	600	3000	1300	7550	360	180	30	240	400	16950	480
5	13	15	13	13	575	12	300	1500	650	3775	180	90	15	120	200	8475	240
6	5	6	5	5	230	5	120	600	260	1510	72	36	6	48	80	3390	120
7	130	150	130	130	5750	120	3000	15000	6500	37750	1800	900	150	1200	2000	84750	1000

For **Table (xlsx) S2** showing the run order to the analytical batches see the appropriate Supplementary Excel (xlsx spreadsheet.

Table S3. The top 25 lipids in the +ve and -ve ESI MS variable importance in projection (VIP) PLS-DA models detected using the discovery LC-IM-MS methodology

	Positive Mode				Negative Mode			
	m/z	RT	CCS	Lipid ID	m/z	RT	CCS	Lipid ID
1	**760.5879**	**4.14**	**305.87**	*** PC(34:1)**	680.6202	6.23	284.58	*Cer(d40:1)
2	**846.7547**	**7.36**	**326.21**	*** TG(50:3)**	694.6357	6.44	287.76	* Cer(d42:1)
3	**850.7874**	**7.58**	**332.91**	*** TG(50:1)**	**766.5401**	**4.48**	**281.74**	***PE(38:4)**
4	**818.7246**	**6.98**	**314.3**	***£ TG(48:3)**	610.5426	4.94	266.89	*£ Cer(d36:1)
5	922.7852	7.32	350.96	* TG(56:7)	**832.6079**	**4.84**	**304.72**	*** PC(36:1)**
6	844.7404	7.04	321.74	* TG(50:4)	695.6393	6.42	288.78	$ Glycinprenol-9
7	**848.7705**	**7.42**	**330.57**	*** TG(50:2)**	764.5243	4.07	280.28	$ PE(38:5)
8	**820.7407**	**7.23**	**318.04**	*** TG(48:2)**	865.5031	1.68	301.28	* PG(44:12)
9	920.7712	7.2	348.43	* TG(56:8)	788.5254	3.83	284.95	* PE(40:7)
10	689.5129	4.55	279.34	$ PA(35:1)	465.3062	1.29	NoID	NoID
11	**758.5723**	**3.75**	**302.37**	*** PC(34:2)**	564.5371	4.95	258.09	*£ Cer(d36:1)
12	788.6181	4.68	308.09	* PC(36:1)	708.6519	6.43		* Cer(d42:1)
13	849.7750	7.51	329.48	$ DG(O-52:4)	**776.5442**	**3.74**	**294.37**	**$ PC(32:1)**
14	810.6024	4.23	318.54	* PC(38:4)	648.6320	6.45	277.93	$ Cer(d42:1)
15	**823.6797**	**6.95**	**313.11**	***£ TG(48:3)**	866.5065	1.68	NoID	NoID
16	577.5199	7.57	267.04	$ DG(O34:3)	620.6017	5.98	270.37	$ Cer(d40:1)
17	659.5218	5.32	274.87	$ TG(36:1	768.5566	4.66	282.53	$ PE(38:3)
18	849.6962	6.99	319.42	$TG(52:7)	610.5425	5.03	266.97	*£ Cer(d36:1)
19	768.5559	4.38	296.79	* PE(18:4)	**804.5779**	**4.28**	**299.62**	*** PC(34:1)**
20	**732.5566**	**3.57**	**294.48**	**$ PC(32:1)**	600.5130	4.96	259.8	$£ Cer(d36:1)
21	**782.5702**	**3.64**	**306.84**	*** PC(36:4)**	762.5104	3.78	279.72	* PE(38:6)
22	813.6864	5.14	319.83	* SM(d42:2)	652.5903	5.73	276.26	$ Cer(d39:1)
23	832.5875	3.52	318.26	* PC(40:7)	746.5147	4.06	278.74	$ PE(O-38:7)
24	904.8338	7.72	346.98	* TG(54:2)	765.5196	3.98	280.4	$ SM(d36:6)
25	549.4882	7.25	258.11	$DG(O-32:3)	367.3590	3.73	202.02	$ FA(24:0)

*Lipids with mass accuracy, mass isotopic pattern score and CCS values within ±3% of prediction

$ Lipids with mass accuracy, mass isotopic pattern score and no predicted CCS values

£ are adducts of the same lipid

BOLD = high VIP scores in both discovery and targeted methods

Database searches on the untargeted data were performed using the LIPIDMAPS database as well as predicted CCS values,(Broeckling et al., 19). A mass error tolerances were 5ppm for the precursor and 10ppm for the fragments with the CCS values within 5% of predicted values was used for the search. Lipostar was used to interrogte these data using a rule-based approach to generate theoretical fragments for lipid structures. A score based on mass error, MS/MS information, and CCS values.is associated with each result and a "traffic light" system visually indicates a level of confidence for identification. Green indicates identifications with high mass accuracy, expected fragment information and high CCS value scores. Amber identifications were based on those with high mass accuracy but may have a sparser MS/MS spectra and no predicted CCS values and thus lower confidence in these identifications. Red indicates lipids with no database hits. To avoid over reporting, we have only reported sum compositions levels for all the lipids in Table S3. As indicated, those assigned * for lipids with mass accuracy, mass isotopic pattern score and CCS values with ±3% of prediction (approved or equivalent to green), the $ was assigned to lipids with good mass accuracy, mass isotopic pattern score but no predicted CCS values (equivalent to amber).

Table S4: Concentrations of gefitinib in tissue extracts and back calculated liver tissue following IV administration at 10 mg/Kg to male mice (3 sig fig)

Time (h)	ng/mL	ng/mg
0.5	14900	300
1	12800	256
3	12100	243
8	3480	70
24	90	2

For **Table (xlsx) S5** showing the quantitative lipid data see the appropriate Supplementary Excel spreadsheet. ((xlsx) S5: Quantitative lipidomic data for liver extracts**)**.

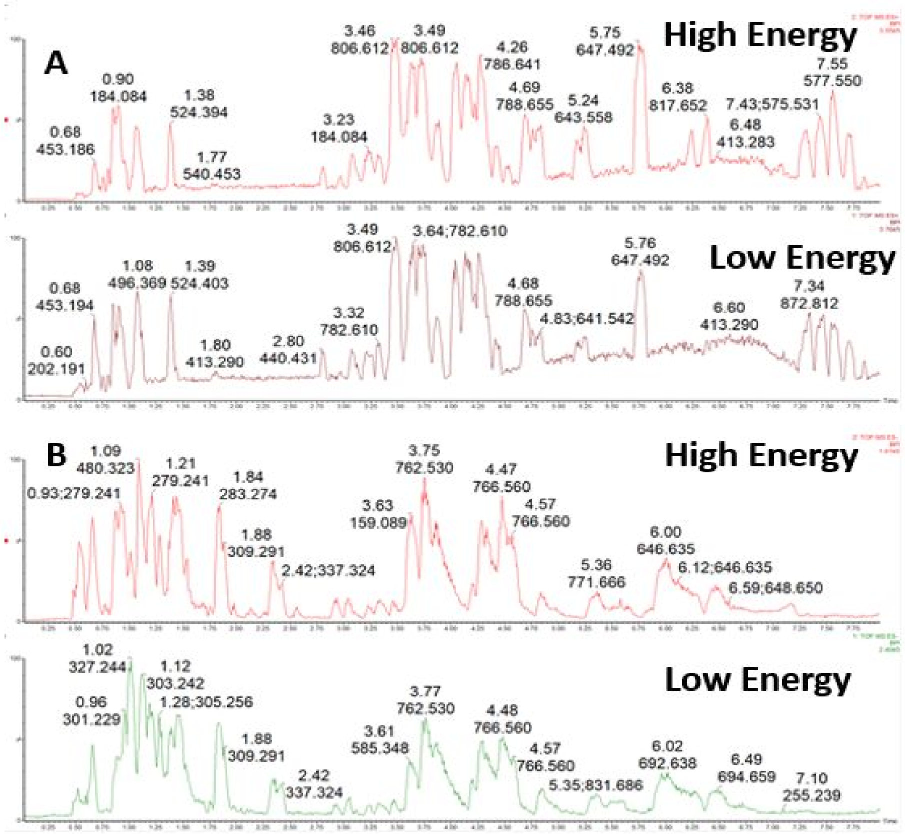

Figure S1: Reversed-phase UPLC-HDMS^e mass chromatograms of mouse liver extracts with (A) +ve ESI and (B) -ve ESI obtained using the discovery LC-IM-MS methodology.

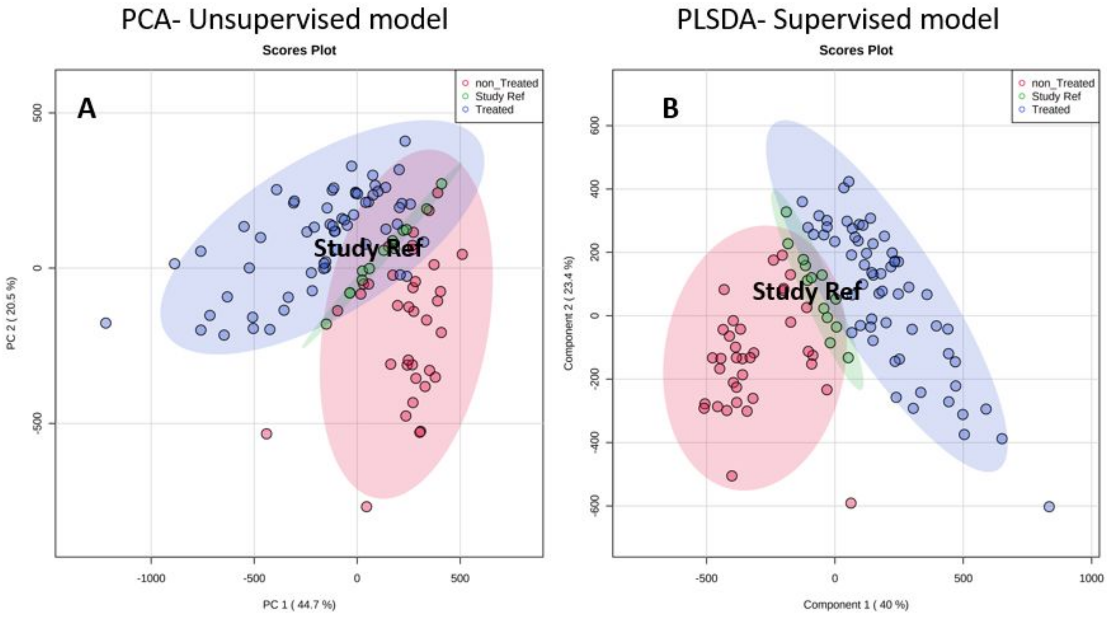

Figure S2: PCA(A) and PLS-DA (B) multivariate statistical model of +ve ESI HDMSe data obtained using the discovery LC-IM-MS methodology, excluding variables present in less than 50% of the pooled study QC samples (standards, gefitinib and metabolites included). Interquartile Range Filter (IQR) Pareto Scaling applied. (treated (blue), matrix control (non-treated (red)) and study ref (green).

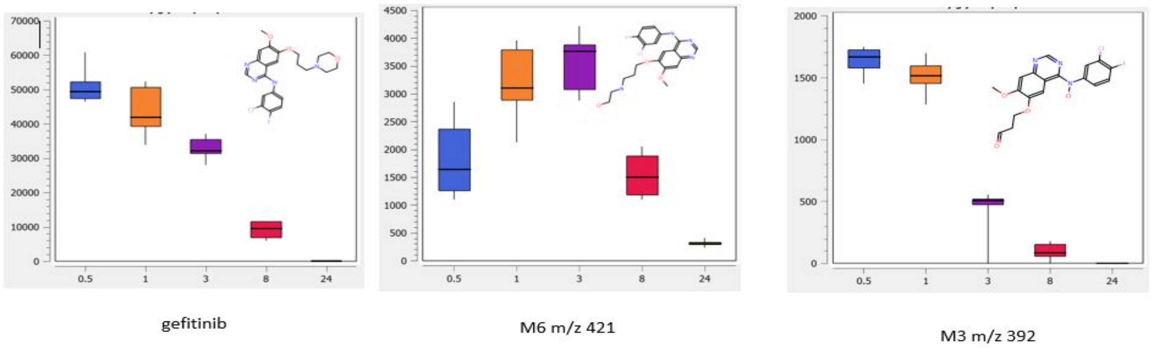

Figure S3. Profiles of gefitinib and its associated metabolites M6 and M3 in the liver lipid extracts following IV administration of the drug at 10 mg/Kg to male mice (+ve ESI-MS).

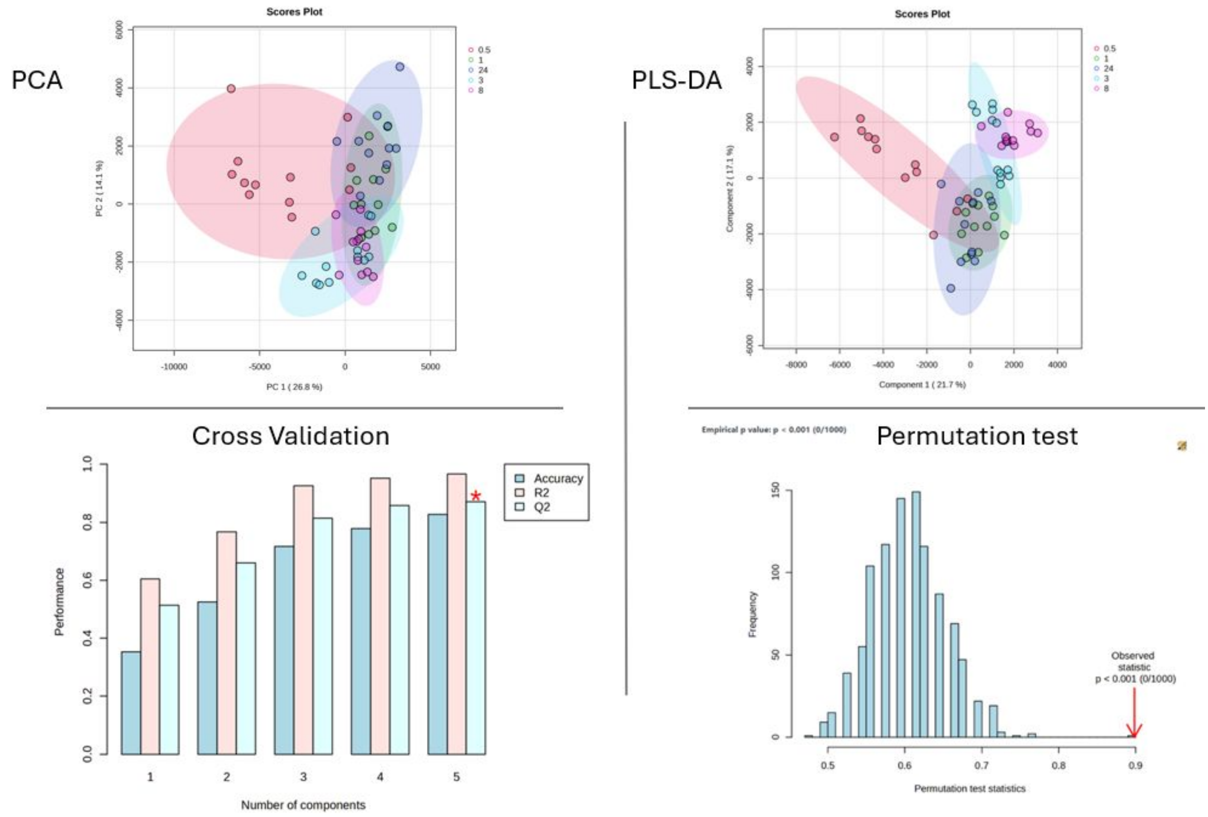

Figure S4. PCA and PLS-DA of RP-UHPLC-IM-MS +ve ESI data (0.5-24 h post dose) data obtained excluding drug-related features and variables in less than 50% of the sample groups. Interquartile range filtering (IQR) and Pareto scaling was applied to generate these models. 5-fold cross-validation (CV 5-fold) of the supervised model showed $R^2 \approx 0.98–1.00$, $Q^2 \approx 0.90–0.92$ and Accuracy ≈ 0.88–0.90. Using 5 components achieved high cross validated performance ($R^2Y \approx 0.95–1.00$, $Q^2 \approx 0.90$, CV accuracy ≈ 0.90). The $R^2–Q^2$ gap was small (<0.1), suggesting limited overfitting. A 1,000 iteration permutation test yielded $p < 0.001$ (0/1000), confirming model performance exceeded that expected under random class assignment.

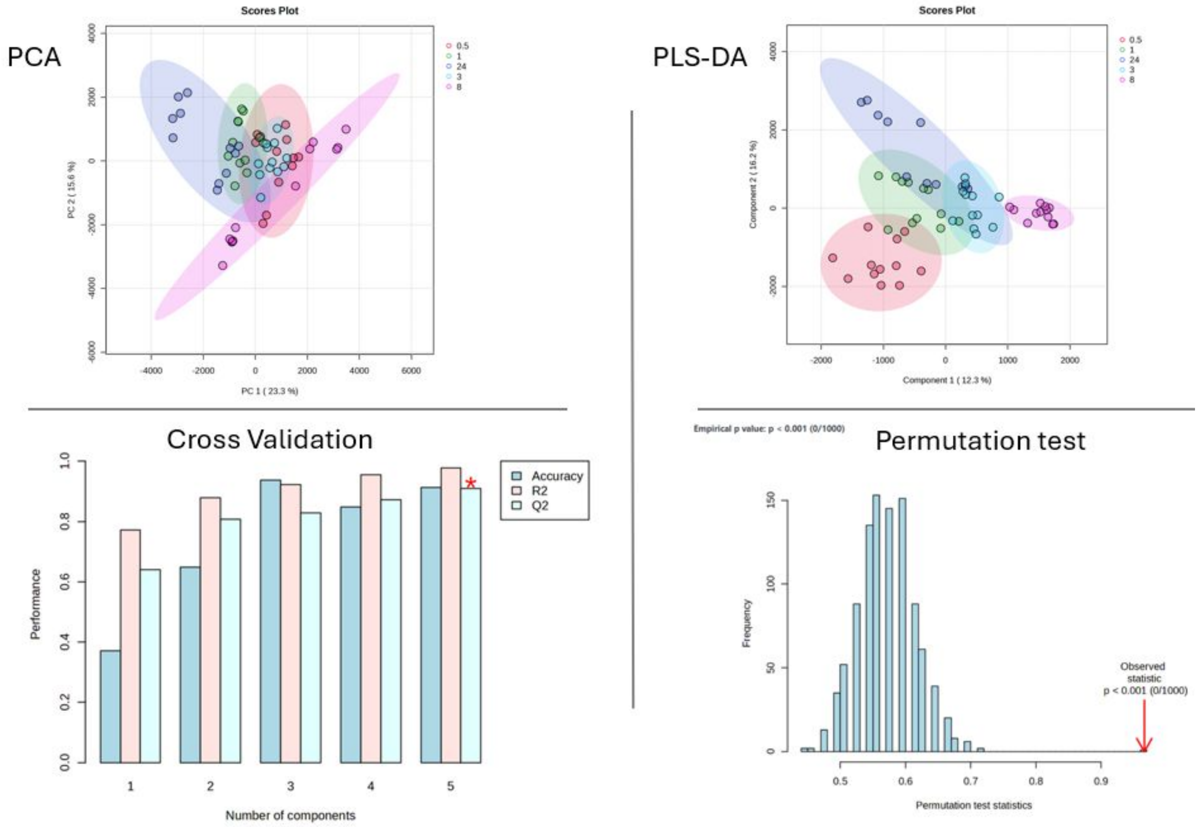

Figure S5. PCA and PLS-DA of RP-UHPLC-IM-MS -ve ESI data (0.5-24 h post dose) excluding drug-related features and variables in less than 50% of the sample groups. Interquartile range filtering (IQR) and Pareto scaling was applied to generate these models. CV 5-fold testing achieved high cross-validated performance with 5 components ($R^2Y \approx 0.98$, $Q^2 \approx 0.90$, CV accuracy ≈ 0.90). The R^2–Q^2 gap was small (<0.1), indicating limited overfitting. A 1000-iteration permutation test showed $p < 0.001$ (0/1000), confirming that model performance exceeded that expected by chance.

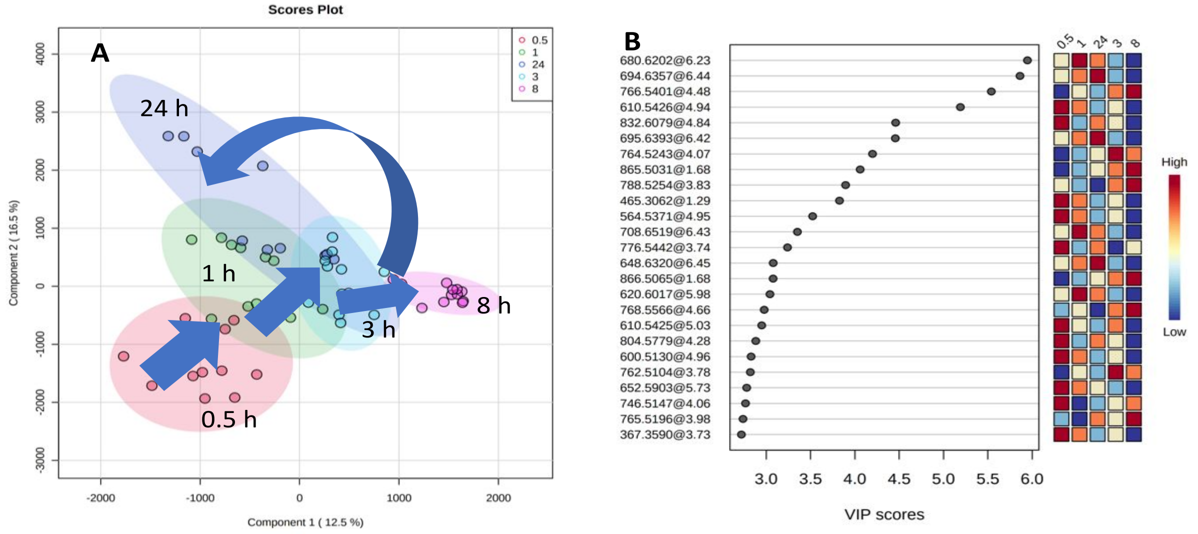

Figure S6: The PLS-DA of RP-UHPLC-IM-MS -ve ESI data shown in Figure S5 A) Score plot and (B) top 25 variable importance in projection (VIP).

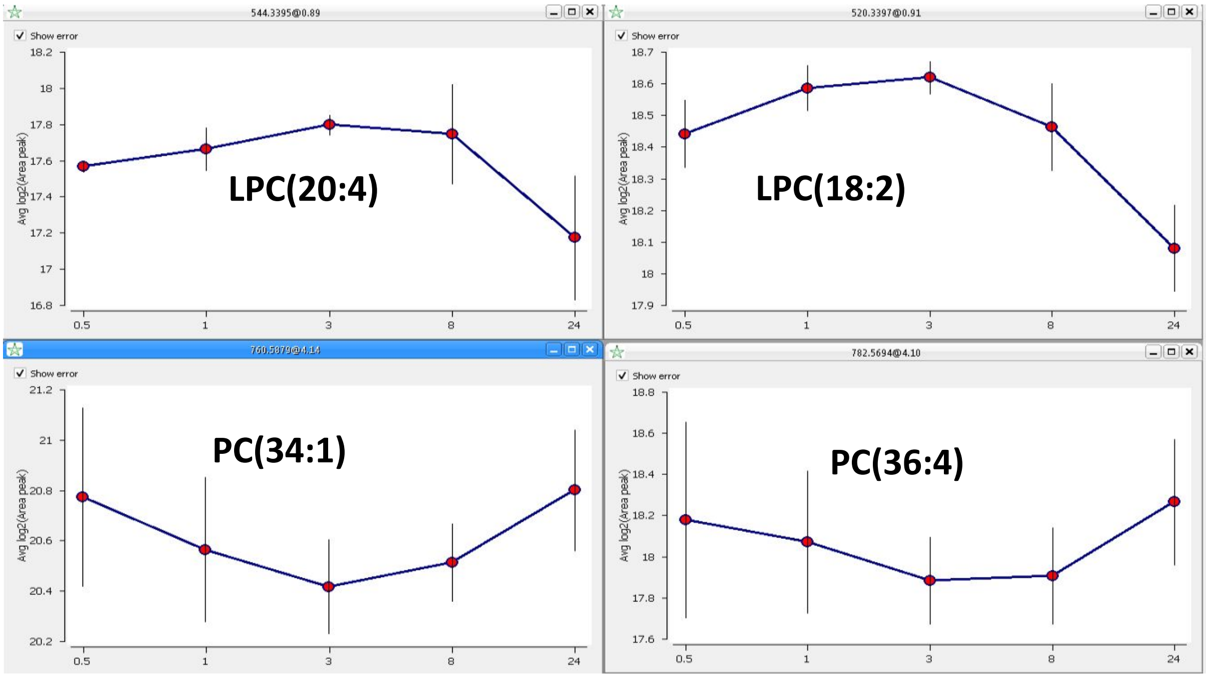

Figure S7: Trend lines for LPCs (20:4) and (18:2) and PCs (34:1) and (36:4) in +ve ESI -MS.

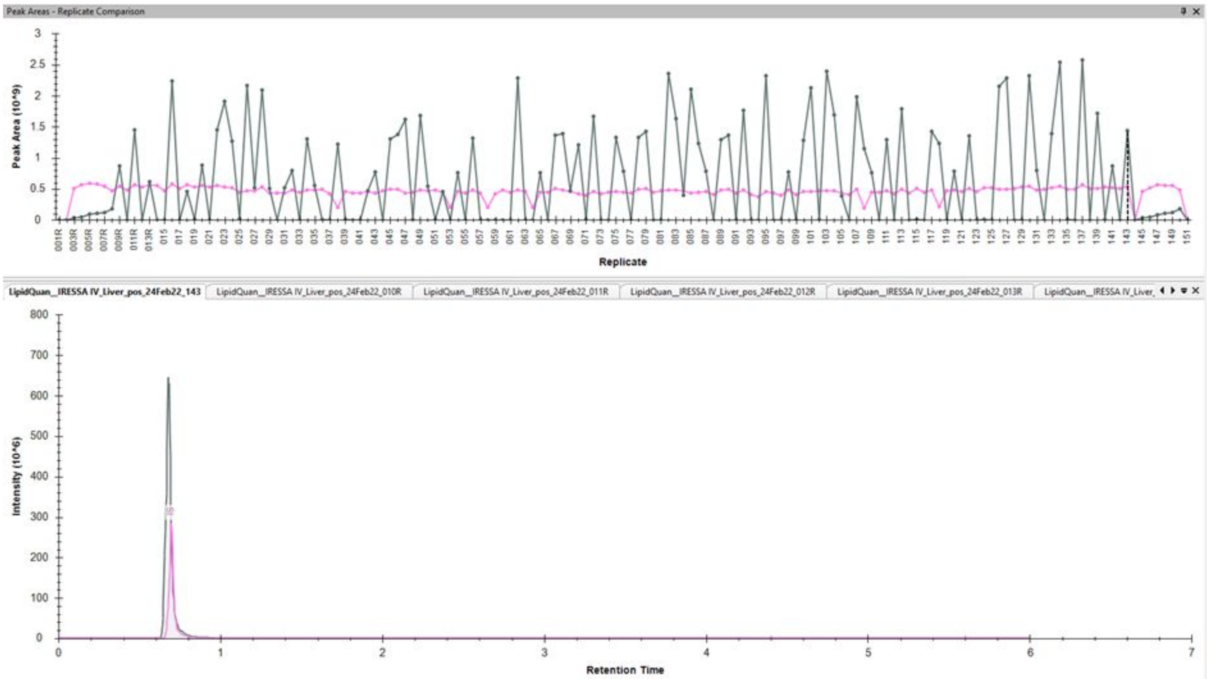

Figure S8 The upper trace shows the responses for gefitinib (grey) and gefitinib d6 (pink) in all of the study samples and QCs whilst the lower trace) shows a typical example for gefitinib (grey) gefitinib-d6 (pink) in a typical study QC.

A

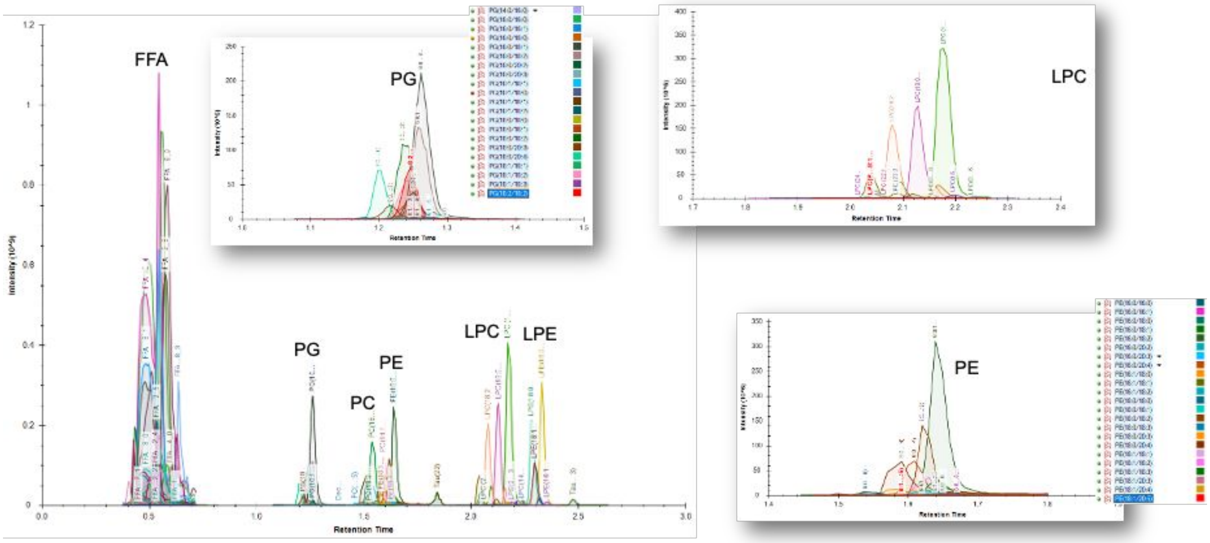

B

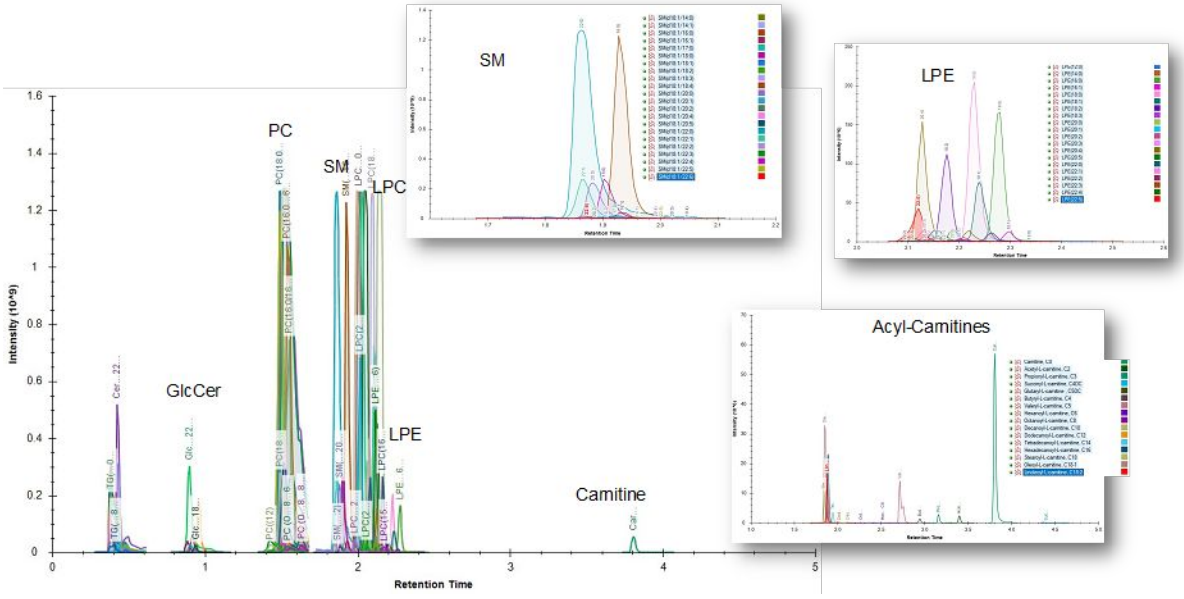

Figure S9. HILIC-based separation of liver lipid extracts using a 2.1 x 100mm ACQUITY BEH Amide 1.7µm column, eluted with a 95% acetonitrile 5% 10 mM ammonium acetate (v/v) Vs 50% ACN, 50% water, 10 mM ammonium acetate (v/v) gradient at 0.6 mL/min over 5 min with -ve (**A**) and +ve ESI MS detection (**B**).

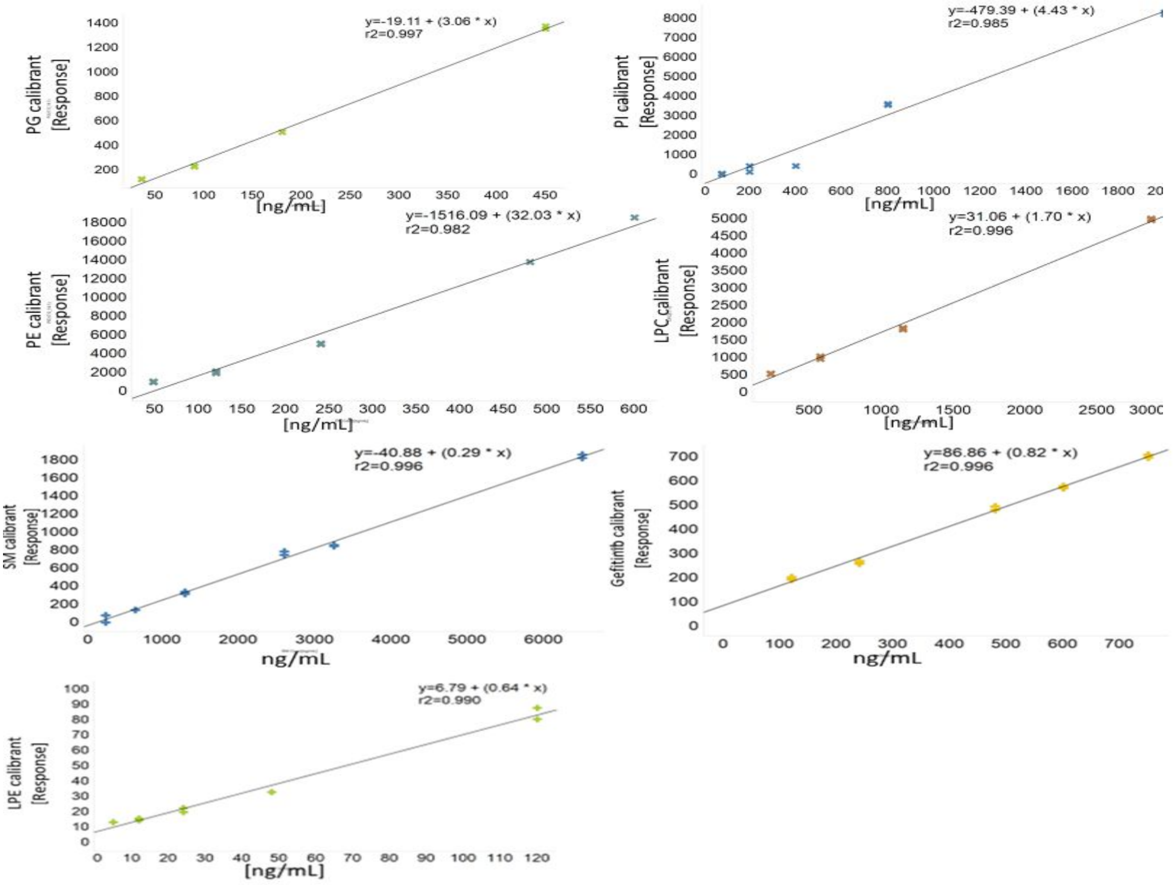

Figure S10: Calibration lines for PG, PI, PE, LPC (-ve ESI MS) and SM, LPE and gefitinib (+ve ESI-MS)

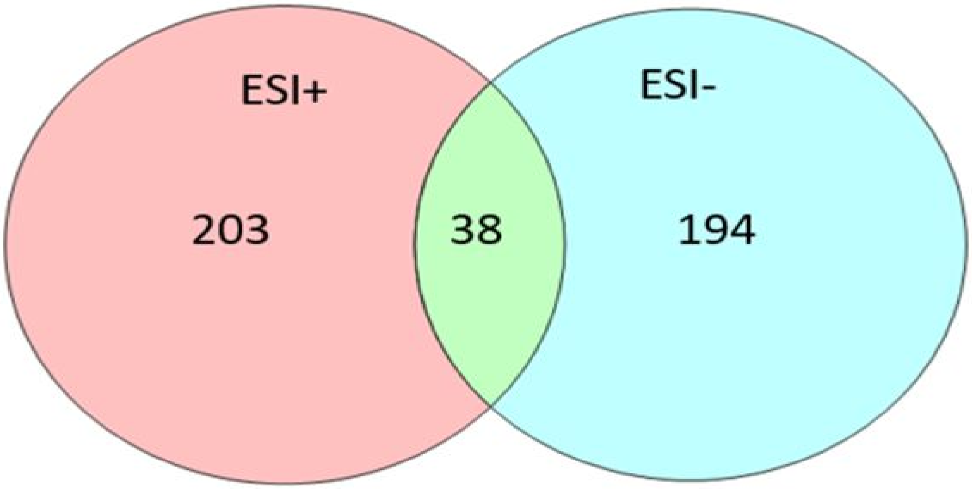

Figure S11. Numbers of lipids monitored by the targeted HILIC-MS method in liver extracts in +ve and -ve ESI showing overlap in those detected using both methods.

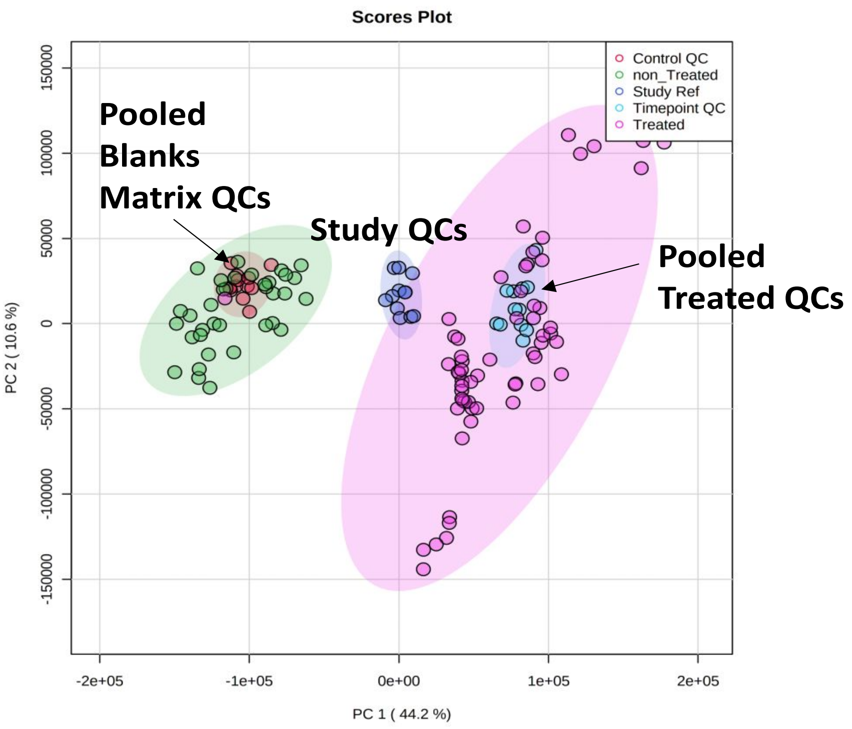

Figure S12: PCA score plot for targeted +ve ESI HILIC-MS data. Variables in less than 50% of samples excludes, standards, gefitinib and metabolites included. Interquartile range filtering (IQR) and Pareto scaling was applied.

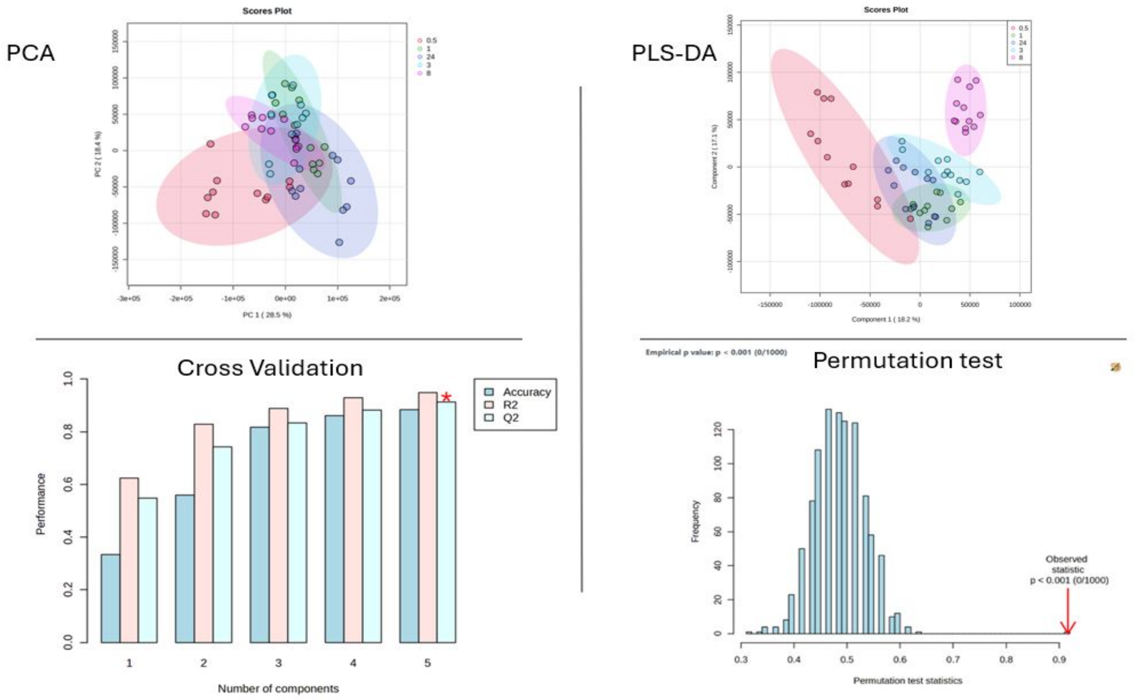

Figure S13. PCA and PLS-DA of +ve ESI HILIC LC-MS/MS +ve ESI data (0.5-24 h post dose) excluding drug-related features and variables in less than 50% of the sample groups. Interquartile range filtering (IQR) and Pareto scaling was applied to generate these models. 5-fold cross-validation (CV 5-fold) achieved high cross-validated performance ($R^2Y \approx 0.95$–1.00, $Q^2 \approx 0.90$, CV accuracy ≈ 0.90). The R^2–Q^2 gap was small (<0.1), suggesting limited overfitting. A 1,000-iteration permutation test yielded $p < 0.001$ (0/1000), confirming model performance exceeded that expected under random class assignment.

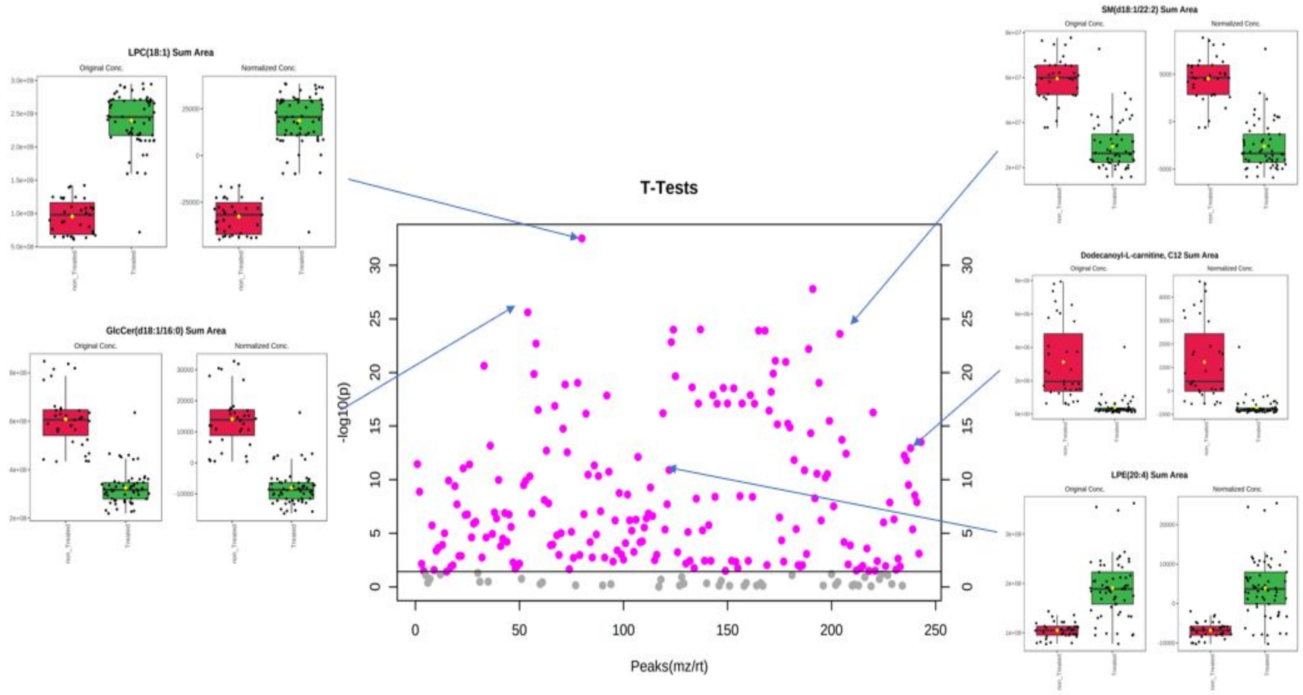

Figure S14 T-Test of targeted +ve ESI MS data, treated (green) vs matrix controls (red). Peak intensity, excluding variables present in less than 50% of pooled study QC samples. Pareto Scaling applied (excludes standards but with gefitinib and its associated metabolites included).

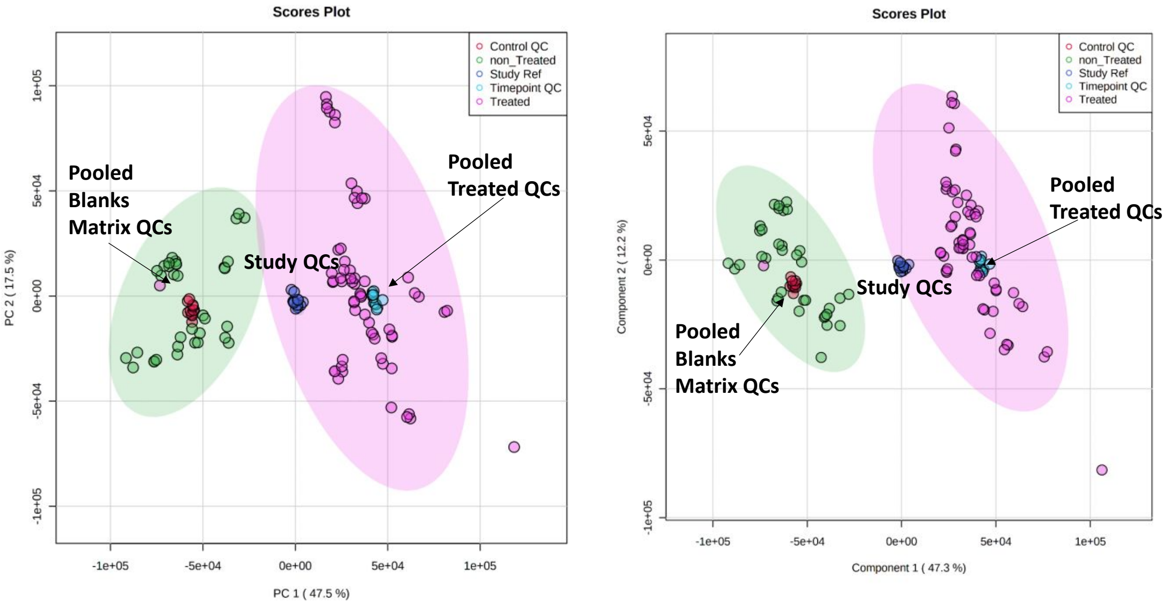

Figure S15: PCA (A) and PLSDA (B) of targeted -ve ESI HILIC-MS data. Peak intensity, excluding variables present in less than 50% of samples, Pareto scaling (standards, gefitinib and its associated metabolites included).

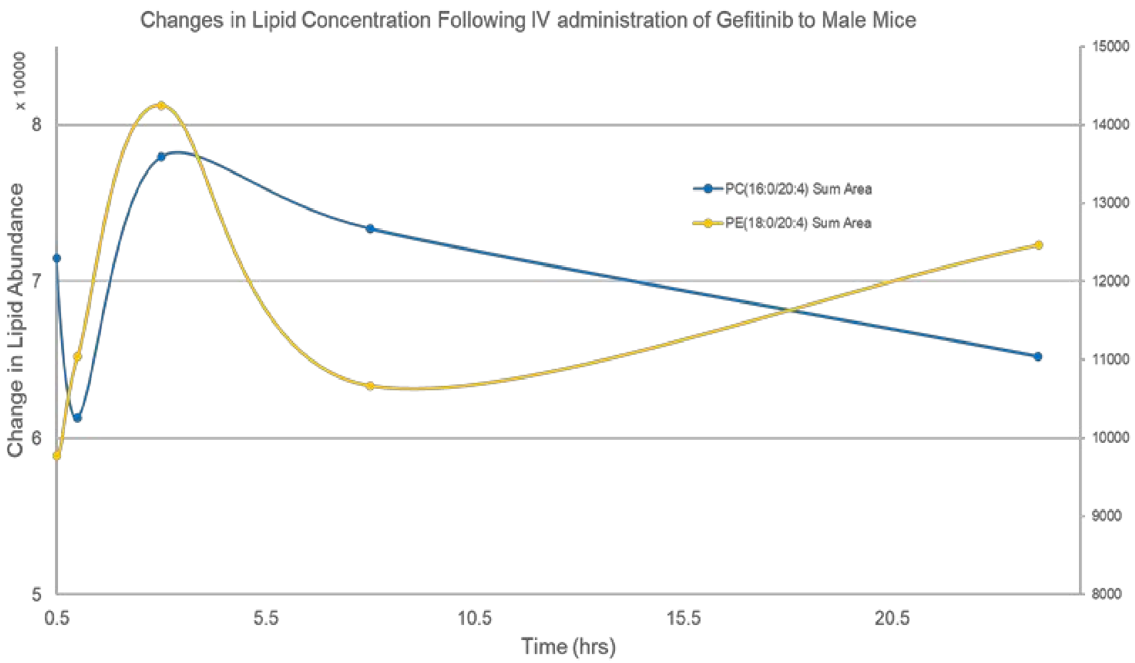

Figure S16: Comparison in changes of lipid abundance following intravenous administration of gefitinib at 10 mg/kg to male mice. -ve ESI HILIC-MS data for PC(36:4), PE(38:4).

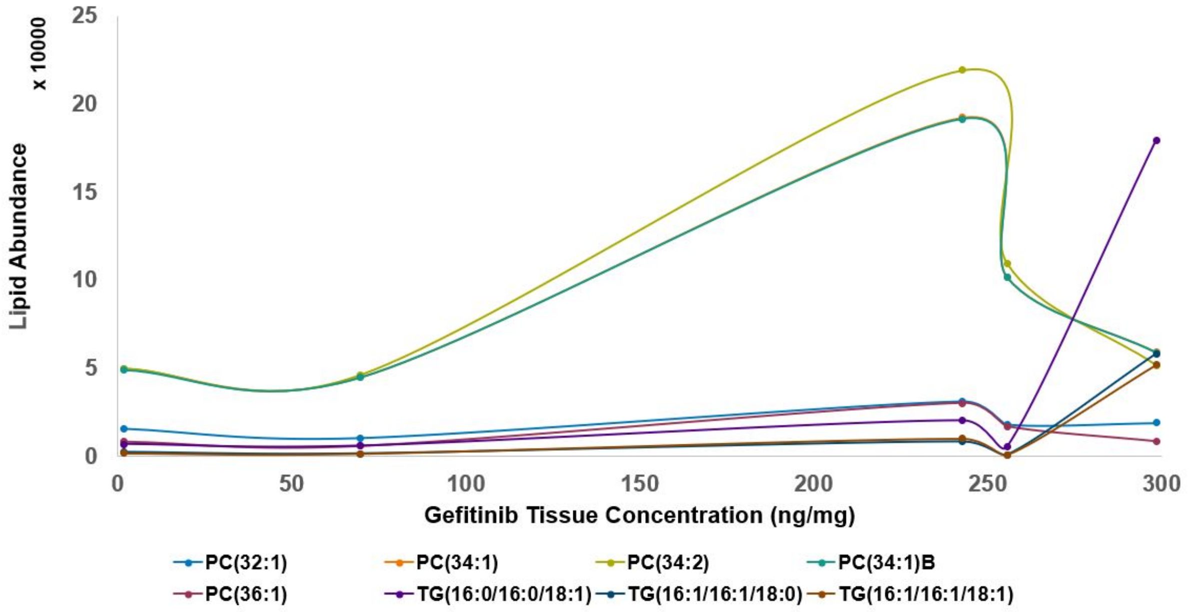

Figure S17: Impact of gefitinib tissue concentrations on selected PC and TG lipid abundances.

A

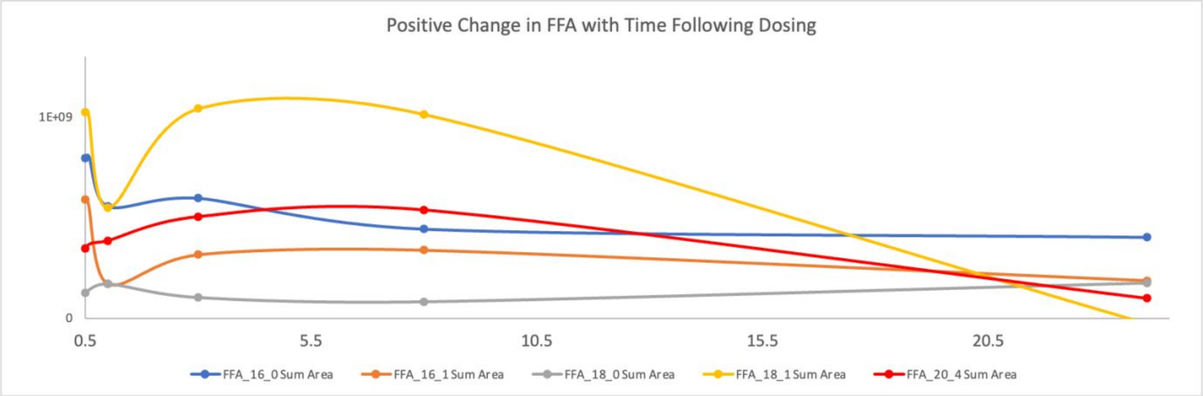

B

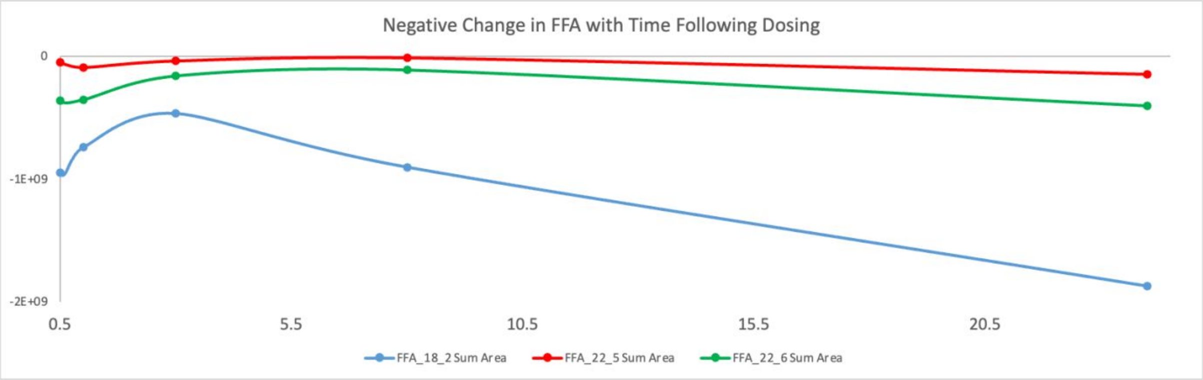

Figure S18: Changes in abundance of selected liver FFA vs time following the IV administration of gefitinib at 10 mg/Kg to mice.

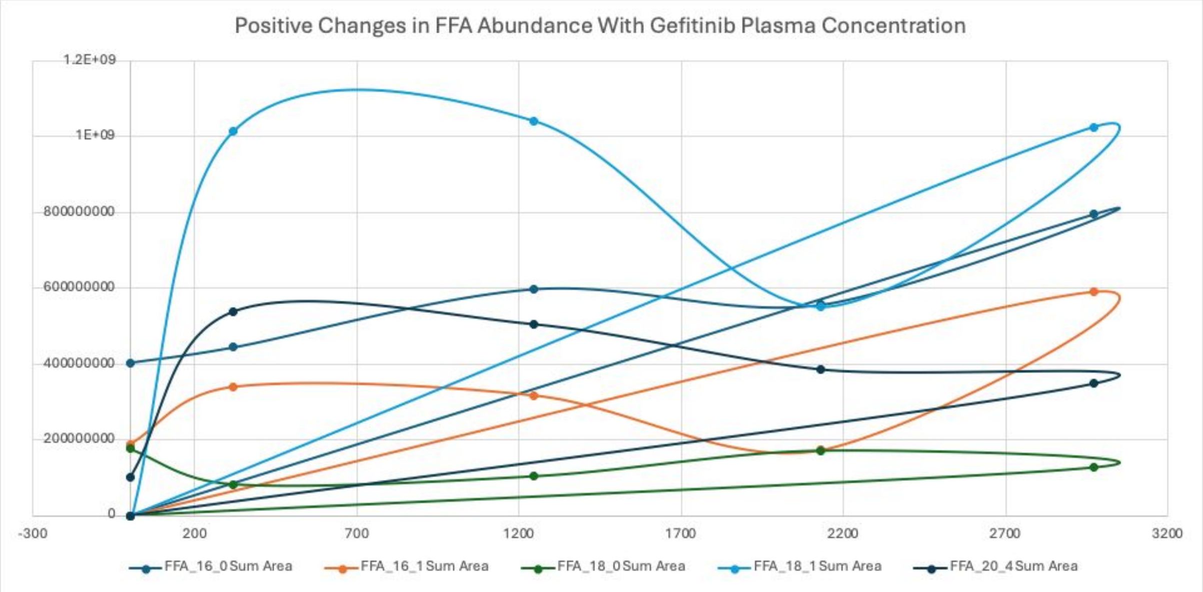

Figure S19: Comparison of FFA abundances in liver extracts with plasma concentrations of gefitinib following IV administration at 10 mg/Kg to mice.

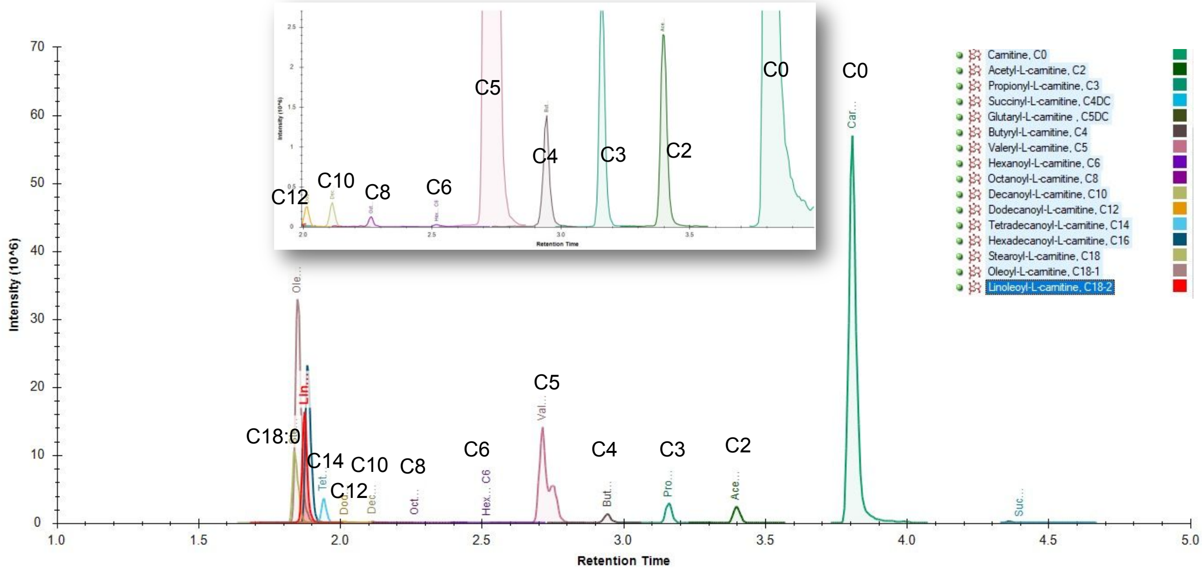

Figure S20: Separation of acyl carnitines (CAR) in mouse liver extracts with detection by +ve ESI MS following the administration of gefitinib at 10 mg/Kg to mice.

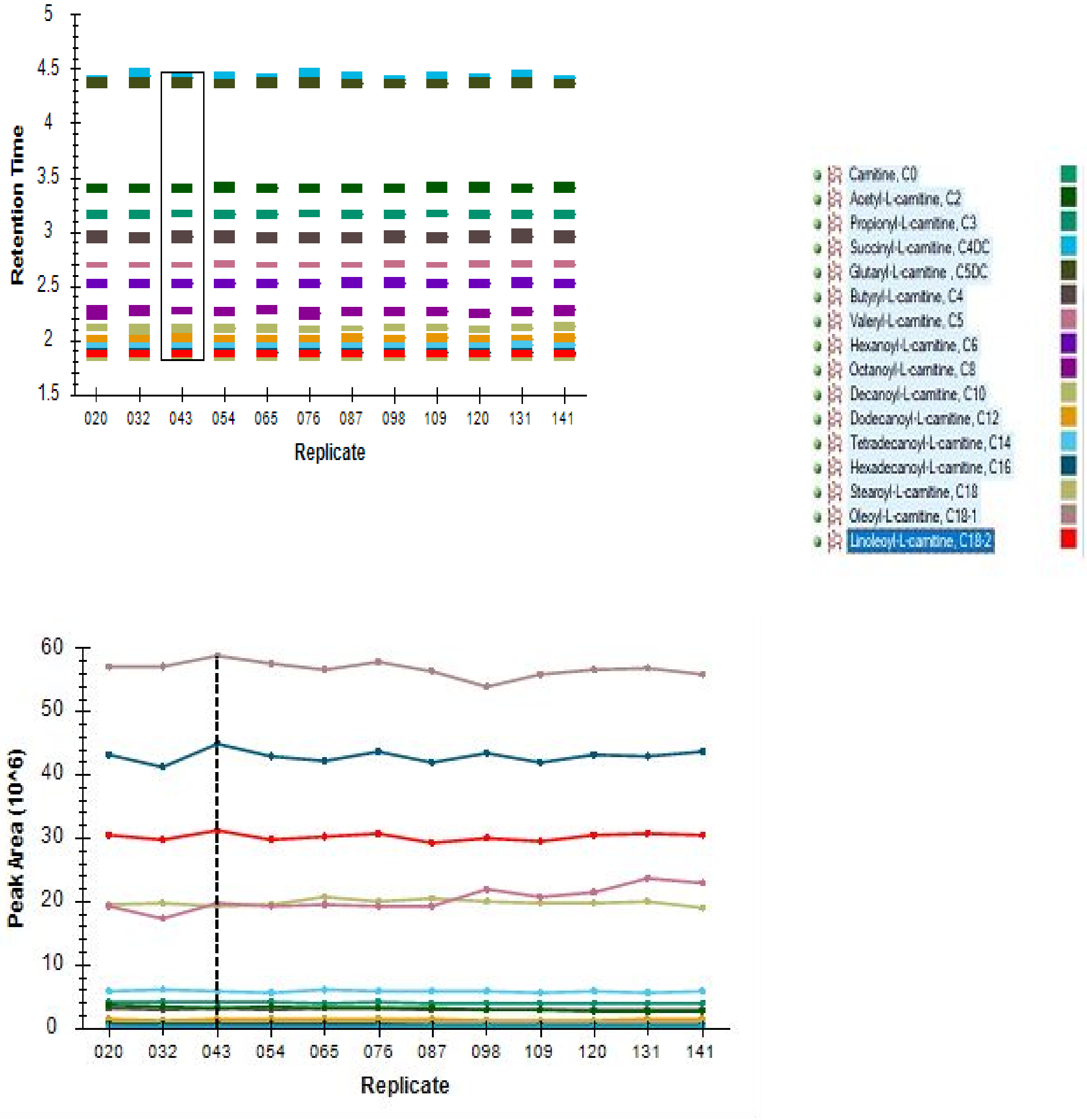

Figure S21: Acyl carnitine t_R (upper) and peak areas (lower) across the randomised sample analysis of liver extracts following IV dosing with gefitinib at 10 mg/Kg to the mouse.

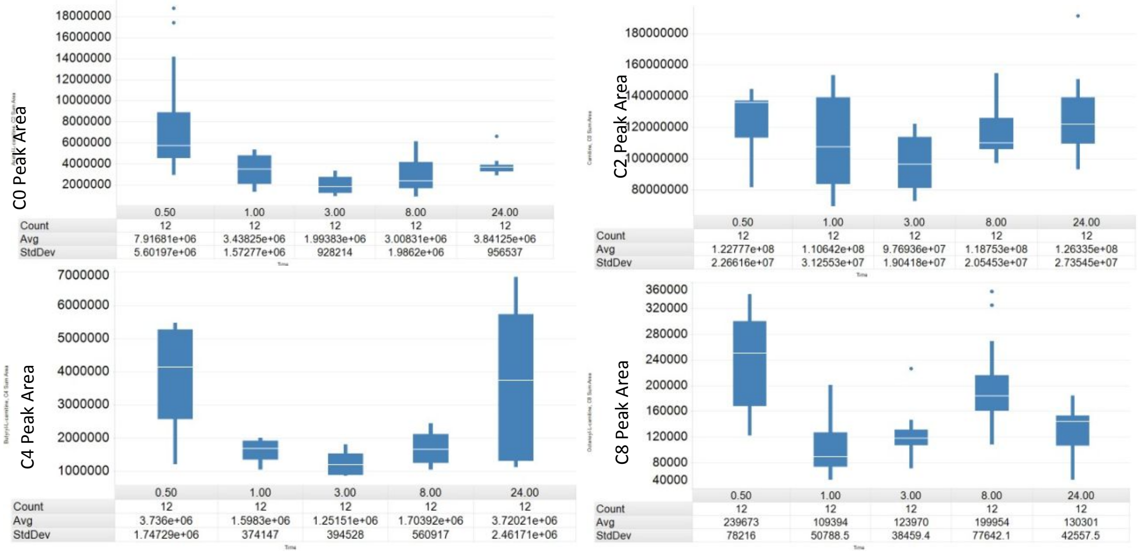

Figure S22: Relative intensity changes for short chain acylcarnitines in mouse livers over time following IV dosing with gefitinib at 10 mg/Kg**.**

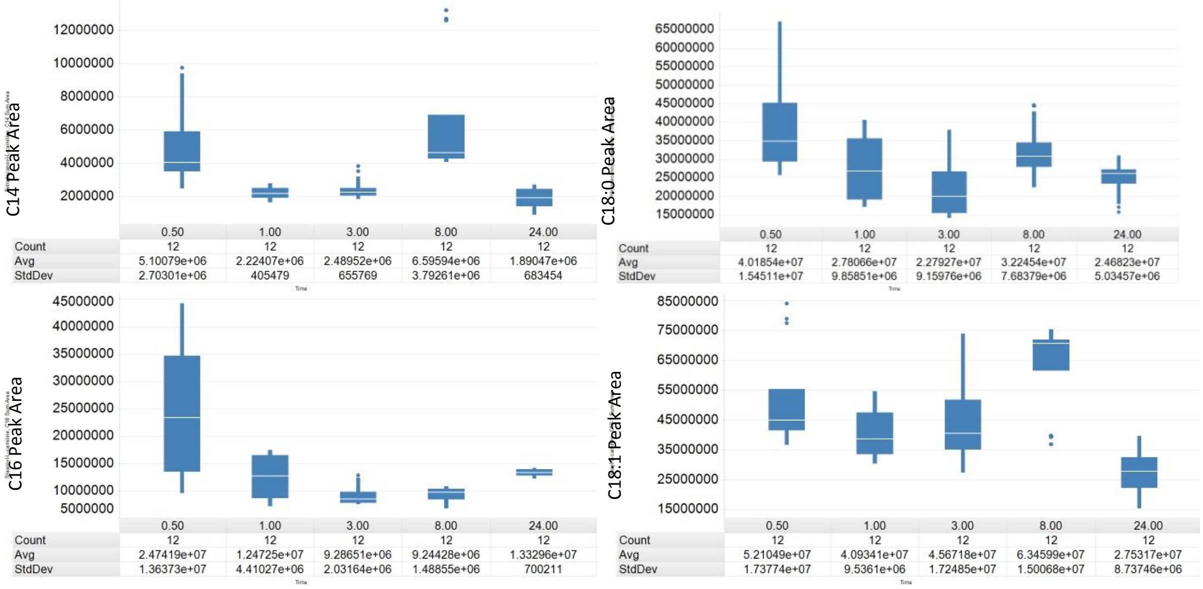

Figure S23: Relative intensity changes in long chain acylcarnitines in mouse livers over time following IV dosing with gefitinib at 10 mg/Kg.
